# Reliable effective number of breeders/adult census size ratios in seasonal‐breeding species: Opportunity for integrative demographic inferences based on capture–mark–recapture data and multilocus genotypes

**DOI:** 10.1002/ece3.3387

**Published:** 2017-10-28

**Authors:** Gregorio Sánchez‐Montes, Jinliang Wang, Arturo H. Ariño, José Luis Vizmanos, Iñigo Martínez‐Solano

**Affiliations:** ^1^ Department of Environmental Biology Universidad de Navarra Pamplona Spain; ^2^ Museo Nacional de Ciencias Naturales, CSIC Madrid Spain; ^3^ Institute of Zoology Zoological Society of London London UK; ^4^ Ecology, Evolution, and Development Group Department of Wetland Ecology Doñana Biological Station, CSIC Seville Spain; ^5^ Department of Biochemistry and Genetics Universidad de Navarra Pamplona Spain; ^6^ Instituto de Investigación en Recursos Cinegéticos (IREC‐CSIC‐UCLM‐JCCM) Ciudad Real Spain

**Keywords:** amphibians, demography, mating system, polygamy, sample size, sibship size prior

## Abstract

The ratio of the effective number of breeders (*N*_b_) to the adult census size (*N*_a_), *N*_b_/*N*_a_, approximates the departure from the standard capacity of a population to maintain genetic diversity in one reproductive season. This information is relevant for assessing population status, understanding evolutionary processes operating at local scales, and unraveling how life‐history traits affect these processes. However, our knowledge on *N*_b_/*N*_a_ ratios in nature is limited because estimation of both parameters is challenging. The sibship frequency (SF) method is adequate for reliable *N*_b_ estimation because it is based on sibship and parentage reconstruction from genetic marker data, thereby providing demographic inferences that can be compared with field‐based information. In addition, capture–mark–recapture (CMR) robust design methods are well suited for *N*_a_ estimation in seasonal‐breeding species. We used tadpole genotypes of three pond‐breeding amphibian species (*Epidalea calamita*,* Hyla molleri,* and *Pelophylax perezi*,* n *=* *73–96 single‐cohort tadpoles/species genotyped at 15–17 microsatellite loci) and candidate parental genotypes (*n *=* *94–300 adults/species) to estimate *N*_b_ by the SF method. To assess the reliability of *N*_b_ estimates, we compared sibship and parentage inferences with field‐based information and checked for the convergence of results in replicated subsampled analyses. Finally, we used CMR data from a 6‐year monitoring program to estimate annual *N*_a_ in the three species and calculate the *N*_b_/*N*_a_ ratio. Reliable ratios were obtained for *E. calamita* (*N*_b_/*N*_a_ = 0.18–0.28) and *P. perezi* (0.5), but in *H. molleri, N*_a_ could not be estimated and genetic information proved insufficient for reliable *N*_b_ estimation. Integrative demographic studies taking full advantage of SF and CMR methods can provide accurate estimates of the *N*_b_/*N*_a_ ratio in seasonal‐breeding species. Importantly, the SF method provides results that can be readily evaluated for reliability. This represents a good opportunity for obtaining robust demographic inferences with wide applications for evolutionary and conservation research.

## 1 INTRODUCTION

The effective size and the census size of a population are two conceptually different demographic parameters. The effective population size (*N*_e_) is a theoretical number that was proposed to measure the strength of inbreeding and genetic drift experienced by finite populations (Crow & Kimura, 1970; Waples, Luikart, Faulkner, & Tallmon, 2013; Wright, 1931). Accordingly, *N*_e_ is defined as the size of an “idealized population” that experiences the same rate of inbreeding or genetic drift as the real population of study (Wright, 1931). As both effects act to reduce genetic diversity, the absolute value of *N*_e_ is directly proportional to the capacity of the population to maintain genetic diversity (Charlesworth, 2009; Ruzzante et al., 2016; Wang, Santiago, & Caballero, 2016; Waples & Antao, 2014). The census size, in contrast, is the total number of individuals in the population or, alternatively, the number of potentially breeding adults (*N*_a_) of the population (Frankham, 1995). Therefore, the ratio between *N*_e_ and *N*_a_ can be considered a measure of the departure from the standard potential of the population to maintain genetic diversity (i.e., *N*_e_ = *N*_*a*_) (Bernos & Fraser, 2016; Frankham, 1995; Palstra & Fraser, 2012; Palstra & Ruzzante, 2008). A high ratio (close to a value of one) suggests that most adults of the population contribute (nearly equally in expectation) to the next generation, approaching the standard scenario of binomial distribution of offspring number per adult. In contrast, a low *N*_e_/*N*_a_ ratio (much smaller than one) implies a strong departure from this standard scenario due to high variance in breeding success among adult individuals, which may potentially lead to genetic impoverishment driven by stochastic processes (Banks et al., 2013).

The *N*_e_/*N*_a_ ratio is strongly dependent on the mating system, which in turn is conditioned by life‐history traits (e.g., longevity, age of sexual maturation and senescence, length of reproductive cycle) and reproductive strategies (e.g., breeding site selection, philopatry, sex‐biased dispersal) that lead to species‐ and sex‐specific constraints to the reproductive investment of individuals (Waples, 2016; Waples et al., 2013). In large or demographically stable populations (with respect to age‐ and sex‐structure), life‐history traits such as the aforementioned can have a major effect on the *N*_e_/*N*_a_ ratio, and therefore, species‐specific *N*_e_/*N*_a_ ratios could be used to estimate *N*_e_ from adult abundance data, or vice versa (Bernos & Fraser, 2016). In contrast, the *N*_e_/*N*_a_ ratio tends to increase in small populations due to genetic compensation mechanisms, and thus variance in the *N*_e_/*N*_a_ ratio among different populations can be informative about microevolutionary processes (Beebee, 2009; Bernos & Fraser, 2016; Palstra & Ruzzante, 2008). Because of the broad informative content of the *N*_e_/*N*_a_ ratio, a large number of studies have addressed its calculation across a wide variety of taxa (Frankham, 1995; Palstra & Fraser, 2012). Community‐based studies focusing on the study of syntopic species with different life‐history traits are especially insightful because they offer a comparative assessment of genetic processes affecting different species in a shared landscape (Fraser et al., 2007; Gomez‐Uchida, Palstra, Knight, & Ruzzante, 2013; Manier & Arnold, 2005, 2006). However, our knowledge about the variation of *N*_e_/*N*_a_ ratios in nature is still limited, because estimation and interpretation of both *N*_e_ and *N*_a_ are challenging (Palstra & Fraser, 2012). In addition, diverse methods are often employed by different researchers to estimate both *N*_e_ and *N*_a_, further complicating comparison among studies.

Direct calculation of *N*_e_ requires comprehensive demographic information (Caballero, 1994; Vucetich & Waite, 1998; Waples, Do, & Chopelet, 2011), and indirect methods (like single‐sample genetic methods) are widely used (Luikart, Ryman, Tallmon, Schwartz, & Allendorf, 2010; Schwartz, Tallmon, & Luikart, 1998; Wang, 2005; Wang et al., 2016). Especially, the linkage disequilibrium (LD) and the sibship frequency (SF) methods have proven the most reliable (Beebee, 2009; Wang, 2016). In species with overlapping generations, estimation of *N*_e_ by these single‐sample methods requires information about the age and sex of the sampled individuals (Nunney, 1993; Wang, Brekke, Huchard, Knapp, & Cowlishaw, 2010; Waples, 2005; Waples & Antao, 2014; Waples, Antao, & Luikart, 2014; Waples et al., 2013), which is often difficult to obtain. However, if all individuals in the genetic sample belong to the same cohort, the effective number of breeding individuals (*N*_b_) producing that cohort can be readily estimated by these methods (Wang, 2009; Waples, 2005; Waples & Antao, 2014; Waples et al., 2013, 2014). Although *N*_b_ retains only part of the information of *N*_e_ (for example, it does not account for age variation in breeding success), it can be used to estimate the ability of the population to maintain genetic diversity (Kamath et al., 2015; Waples, 2005; Waples & Antao, 2014; Waples et al., 2013). Furthermore, estimates of *N*_b_ obtained across successive breeding seasons can be used to calculate *N*_e_ (Whiteley, Coombs, O'Donnell, Nislow, & Letcher, 2017). Thus, the *N*_b_/*N*_a_ ratio can be considered an approximation of the effective/census size ratio as applied to a single breeding season.

Although some methods based on direct counts, acoustic surveys, or extrapolations from evidences of breeding activity have been employed to estimate *N*_a_, individual‐based capture–mark–recapture (CMR) methods provide the most accurate insights about population size variation (Clutton‐Brock & Sheldon, 2010). Capture–mark–recapture studies are time‐consuming, but current techniques include a wide range of sophisticated sampling designs that can be applied to different types of data (Lebreton, Burnham, Clobert, & Anderson, 1992; Tavecchia, Besbeas, Coulson, Morgan, & Clutton‐Brock, 2009). In particular, robust design frameworks, which rely on nested CMR sessions, are especially powerful for *N*_a_ estimation (Kendall, Pollock, & Brownie, 1995; Pollock, 1982). The efficiency of robust design analyses can be maximized when the capture of individuals is concentrated in short time periods, during which population closure can be assumed (Kendall & Nichols, 1995; Kendall, Nichols, & Hines, 1997). This is the case of seasonal‐breeding species, in which adult individuals congregate during a few weeks every year (e.g., in lekking aggregations); this allows the concentration of sampling sessions, facilitating annual estimation of *N*_a_, corresponding to the number of sexually mature individuals which are seeking to reproduce.

In this regard, pond‐breeding amphibians in temperate latitudes represent an excellent study system because their seasonal aggregative breeding behavior facilitates annual *N*_a_ estimation by robust design CMR methods (Cayuela et al., 2014; Cayuela, Arsovski, et al. 2016; Cayuela, Boualit, et al. 2016; Muths, Scherer, & Bosch, 2013). Similarly, the spatial and temporal clustering of tadpoles of the same cohort in the breeding sites makes them especially suitable for *N*_b_ estimation (Beebee, 2009; Wang, 2009; Waples, 2005). The SF method, implemented in colony (Jones & Wang, 2010; Wang, 2009), is especially convenient for *N*_b_ estimation in seasonal‐breeding species. This method has proved accurate for *N*_b_ estimation when sample size is close to, or higher than, real *N*_b_ (Ackerman et al., 2017; Beebee, 2009; Wang, 2016), or when highly informative markers are used. In addition, the SF method is based on sibship reconstruction, which provides demographic inferences (e.g., the total number of mating pairs or the average number of partners for individuals of each sex) that can be compared with evidences of breeding activity such as egg string counts, direct mating observations, or individual records of permanence in the breeding sites. This calibration with field information allows the supervision of reconstructed sibship relationships, on which the estimate of *N*_b_ is based. Furthermore, inclusion of genotype information of candidate sires and dams potentially increases the robustness of sibship reconstruction, thereby improving *N*_b_ estimates. Finally, replicated analyses varying the analytical settings and the genetic information employed (i.e., the numbers of sampled markers and individuals) can be used to check for the convergence of results.

Demographic approaches integrating SF estimation of *N*_b_ and CMR estimation of *N*_a_ provide a good opportunity for producing reliable *N*_b_/*N*_a_ ratios for seasonal‐breeding species, such as pond‐breeding amphibians of temperate latitudes. Here, we implemented such an integrative study, combining field‐based information with genotype data from newly optimized sets of microsatellite markers to monitor a breeding assemblage of three sympatric anuran species differing in their life‐history traits: the natterjack toad *Epidalea calamita* (Laurenti, 1768); the Iberian treefrog *Hyla molleri* Bedriaga, 1889; and Perez's frog *Pelophylax perezi* (López‐Seoane, 1885). *Epidalea calamita* is an explosive breeder that selects ephemeral sites for laying eggs, taking advantage of their fast‐developing tadpoles, whereas *H. molleri* and *P. perezi* are characterized by longer breeding seasons and larval stages (García‐París, Montori, & Herrero, 2004). A prolonged breeding season is usually associated with increased polygamy rates and a higher number of successfully breeding individuals in the population (in the absence of strong intrasexual competition; Byrne & Roberts, 2012). As a consequence, species with longer breeding seasons are expected to show higher *N*_b_/*N*_a_ ratios. In addition, population‐specific processes, such as genetic compensation mechanisms in small populations, can also increase *N*_b_/*N*_a_ ratios. Here, we discuss the potential role of these factors, specifically aiming at:

1. Estimating annual *N*_a_ (number of adult males and females separately) of the three species in a breeding locality using CMR robust design methods.
2. Estimating *N*_b_ of the three species using the SF method and assessing the reliability of these estimates by comparing reconstructed families with independent information about each species’ phenology and evidences of breeding activity, and by checking for the consistency of results in replicated analyses with different priors, number of markers, and sample sizes.
3. Calculating the corresponding *N*_b_/*N*_a_ ratio for each species.

## 2 MATERIALS AND METHODS

### 2.1 Study area and CMR monitoring program

Our area of study comprises the vicinities of *Laguna de Valdemanco*, a temporary aquatic system that extends across a maximum surface area of 12,800 m^2^ (when adjacent meadows are flooded in early spring), with one meter of maximum depth. This pond is located in the foothills of *La Cabrera* ridge, 1,055 m above sea level, between the towns of *La Cabrera* and *Valdemanco* (Madrid, Spain). It is surrounded by Mediterranean shrubland dominated by gum rockrose (*Cistus ladanifer*). We carried out a 6‐year monitoring program of the amphibian community at this locality between 2010 and 2015, with CMR sessions performed every year in *Laguna de Valdemanco* and in some additional minor breeding sites at a distance between 270 and 800 m from the main pond (Sánchez‐Montes & Martínez‐Solano, 2011). Six amphibian species breed regularly in the pond (*Pleurodeles waltl*,* Triturus marmoratus*,* Pelobates cultripes*,* Epidalea calamita*,* Hyla molleri,* and *Pelophylax perezi*), and dispersive individuals of *Alytes cisternasii, Bufo spinosus,* and *Discoglossus galganoi* were also recorded occasionally. We addressed the estimation of the *N*_b_/*N*_a_ ratio for *E. calamita*,* H. molleri,* and *P. perezi*, three of the species for which CMR work proved most successful, based on the recapture rates obtained.

The annual number of egg strings of *E. calamita* was also recorded. Among the three targeted species, this is the only one that lays clutches mainly in shallow water, thus allowing exhaustive counts (García‐París et al., 2004). Counts were performed every year during the whole *E. calamita* breeding season, from the appearance of the first strings to the end of the mating period, when the puddles and shallow areas selected by this species for egg‐laying finally dried up. All shores and shallow areas (<0.5 m deep) were inspected visually 3–6 times during each breeding season; egg strings of *E. calamita* were counted, their position was recorded, and their development was monitored in subsequent visits to avoid overestimation.

### 2.2 CMR estimates of *N*_a_

As part of the monitoring program, nocturnal CMR sessions were performed during the breeding season of each species every year from 2010 to 2015. The entire water surface of *Laguna de Valdemanco*, shores, and nearby areas was sampled on foot without time limit, in order to maximize the number of captures. Adult individuals were captured by hand or with the help of dip nets, sexed based on external morphological features and marked with an 8 mm AVID M.U.S.I.C transponder (EzID, Greeley, Colorado, USA), with a unique identity code readable with an AVID Minitracker II device. Three phalanges of a toe of every marked individual were clipped and stored in absolute ethanol for genetic analyses. Toe clipping in these three species did not affect survival of individuals, as suggested by the observed rapid healing (see also McCarthy & Parris, 2004). Bone samples were also used for skeletochronological studies (Sánchez‐Montes et al., unpublished data). All individuals were released back in their place of capture after processing.

Capture–mark–recapture sessions were planned to fulfill the assumptions of the robust design method (Pollock, 1982), by minimizing the time span among secondary sampling occasions (in this case, within each breeding season, with a median time span of 11 days), relative to the time span between primary samples (in this case, between different years). Our final CMR datasets included capture histories for 542 adult *E. calamita* (141 females, 401 males), 415 adult *H. molleri* (57 females, 358 males), and 190 adult *P. perezi* (94 females, 96 males) marked between 2010 and 2015. The total number of captures was 1,512 for *E. calamita* (1–17 captures per individual in 26 total CMR sessions), 526 for *H. molleri* (1–4 captures per individual in 17 sessions), and 312 for *P. perezi* (1–6 captures per individual in 19 sessions). Return rates (the proportion of individuals captured more than once) were 0.58, 0.23, and 0.41 for *E. calamita, H. molleri,* and *P. perezi*, respectively.

We analyzed interannual variation in *N*_a_ using the robust design method implemented in mark (White & Burnham, 1999). Different models were generated by applying constraints (time and/or sex dependence) to annual survival (S). As no time limit was imposed to standardize capture effort in the CMR sessions, individual probability of capture was always modeled as dependent of sex and time. The probability of capture was set equal to the probability of recapture in all models (i.e., we did not introduce a trap‐dependence factor in any model). As no optimum goodness‐of‐fit tests have been proposed for robust design models, we tested for the most common causes of departures from the Cormack–Jolly–Seber (CJS) model assumptions among secondary occasions (Schwarz & Stobo, 1997). We used U‐CARE (Choquet, Lebreton, Gimenez, Reboulet, & Pradel, 2009) to test for “transience” and “trap‐dependence” effects in each year in which the required minimum number of CMR sessions were available: a minimum of three CMR sessions are necessary to test for “transience” and four CMR sessions are necessary to test for “trap‐dependence.” Thus, 4 years were suitable for analysis in the case of *E. calamita*, 2 years in *H. molleri,* and 3 years in *P. perezi*. We tested different models assuming that the probability of temporary emigration/immigration was either (1) dependent of the last probable state of the individual (Markovian), (2) independent of the last probable state of the individual (random), or (3) absent (i.e., temporary emigration/immigration fixed to zero). These temporary immigration/emigration probabilities could reflect actual temporary displacements out of the area of study or individuals skipping a breeding season (i.e., interannual changes in state between “breeder” and “non‐breeder,” Cayuela et al., 2014; Cayuela, Arsovski, et al. 2016; Cayuela, Boualit, et al. 2016; ; Muths, Scherer, Corn, & Lambert, 2006; Muths et al., 2013). Models for estimation of *N*_a_ were ranked based on the Akaike information criterion, corrected for small sample sizes (AICc, Akaike, 1974; Burnham & Anderson, 2002), and estimates of *N*_a_ were obtained by weighted averaging estimates from the candidate models.

### 2.3 Genetic estimates of *N*_b_

We used adult and single‐cohort tadpole genotypes for *N*_b_ estimation. Tadpole genotypes included two samples for *E. calamita*, collected in the breeding seasons of 2013 and 2015 (*n *=* *77 and 73 tadpoles, respectively), one for *H. molleri* in 2013 (*n *=* *96), and one for *P. perezi* in 2010 (*n *=* *94, Table 1). Microsatellite DNA genotypes of tadpoles of *E. calamita* (2013), *H. molleri,* and *P. perezi* were obtained from Sánchez‐Montes, Ariño, Vizmanos, Wang, and Martínez‐Solano (2017), and we genotyped an additional single‐cohort sample of tadpoles of *E. calamita* in 2015 (Table 1). Tadpoles were sampled in a comprehensive survey across the entire surface of *Laguna de Valdemanco* and in a small area of adjacent flooded meadows, which together comprise a single, continuous breeding site (Sánchez‐Montes & Martínez‐Solano, 2011; Sánchez‐Montes et al., 2017). We also used a subsample of the tissue collection obtained during the 6‐year monitoring program as candidate parents for SF analyses (Table 1). This subsample included adult males and females that had been captured in the area of study within a period from 1 year before to 1 year after the breeding season when tadpoles were collected (including both the 2013 and 2015 breeding seasons in the case of *E. calamita*, see Table 1). All individuals were genotyped using three sets of 15–17 polymorphic microsatellites specifically designed for each species following the methods described in Sánchez‐Montes, Recuero, Gutiérrez‐Rodríguez, Gomez‐Mestre, and Martínez‐Solano (2016), Sánchez‐Montes et al. (2017). Basic properties of the three sets of markers and genetic diversity estimates obtained in *Laguna de Valdemanco* can be found in Sánchez‐Montes et al. (2017).

**Table 1:** Sample sizes (*n*) in SF analyses (genotypes obtained from Sánchez‐Montes et al. (2017) are indicated with an asterisk “*”) and estimates (with 95% CIs) of *N*_b_ and *N*_a_ obtained for each species. Also, the total number of sires and dams inferred in SF analyses (in parentheses, the number of inferred parents included in the genotyped samples of candidate parents) is shown for each species, along with the egg string counts for *E. calamita*. *N*_b_/*N*_a_ was calculated by dividing the point SF estimate of *N*_b_ by the sum of *N*_a_ point estimates for males and females in each species (total *N*_a_). Nonestimable parameters are indicated with “–”

Species	Year	*n*			*N*_b_	*N*_a_			Inferred number of sires	Inferred number of dams	*N*_b_/*N*_a_	Egg string counts
		Tadpoles	Males	Females		Males	Females	Total				
*E. calamita*	2013	77*	198	102	51 (35–78)	138 (133–143)	43 (28–58)	181	23 (15)	23 (16)	0.28	46
	2015	73			52 (35–80)	162 (158–165)	125 (78–172)	287	31 (27)	29 (22)	0.18	104
*H. molleri*	2013	96*	48	48	131 (97–179)	126 (102–150)	–	–	52 (18)	51 (5)	–	–
*P. perezi*	2010	94*	47	48	69 (49–98)	69 (30–108)	68 (4–133)	137	37 (17)	38 (24)	0.50	–

We used tadpole and adult genotypes of each species to reconstruct sibship and parentage and to obtain estimates of *N*_b_ based on SF analyses in colony Version 2.0.6.1 (Jones & Wang, 2010). We calculated the probability that the progenitors of the offspring samples were among the genotyped adult individuals using *N*_a_ estimates from CMR analyses, by dividing the sample size of candidate fathers (mothers) of each species by the estimated *N*_a_ of males (females) in the corresponding year. As no estimate of *N*_a_ was available for females of *H. molleri*, we used the same probability as for males (i.e., 48/126 = 0.38). We also performed additional analyses with different probabilities of parents being present in the genotyped samples (0.5 for both sexes of *E. calamita* and 0.2 for *H. molleri* and *P. perezi*) to check for the dependence of results on these prior probabilities. Based on previously estimated error rates (Sánchez‐Montes et al., 2017), we used a genotyping error rate of 0.05 for every marker in *E. calamita* and of 0.01 in each of the remaining two species. As offspring samples represent a single year cohort, the assumption of the mating system of the species required for colony analyses refers to the possibility of multiple matings *within* a single breeding season. Low rates of double‐clutching females have been reported in some *E. calamita* populations in Sweden and UK (Denton & Beebee, 1996; Silverin & Andrén, 1992) and double clutching and multiple clutching have been observed in some *Hyla* species in Europe (Broquet, Jaquiéry, & Perrin, 2009; Cadeddu & Castellano, 2012). However, it is unknown whether females of *H. molleri* and *P. perezi* lay more than one clutch per year (sequential polyandry) or whether there are multiple paternities within each clutch (simultaneous polyandry), although the former scenario seems more likely (Byrne & Roberts, 2012; Lengagne & Joly, 2010). Accordingly, we conservatively performed all analyses by assuming polygamy in both sexes in the three species, with “very long” run length and “very high” precision settings.

### 2.4 Sensitivity of *N*_b_ estimates to sibship size prior, sample size, and number of loci

We explored the effects of using different sibship size priors, number of markers, and sample sizes on sibship and parentage reconstruction and on *N*_b_ estimates. First, we compared the effect of using different sibship size priors from an average sibship size of one up to five, or not using any prior information. The average sibship size is the mean number of offspring sired by each breeding male (paternal sibship size) and female (maternal sibship size). Setting a low average value for both sexes (i.e., = 1) may help discourage false full and half‐sib assignments, improving *N*_b_ estimation, when marker information is insufficient (Wang, 2016). High average values are only expected in samples obtained from a low number of potential breeders or in cases of strong male (or female) dominance. Second, we explored the effect of marker information by performing jackknifed replicates for each number of loci in each species dataset, from one locus to the complete set, either using or not using a prior sibship size = 1. Third, we performed replicates at different‐sized jackknifed offspring (tadpole) subsamples (but using the complete candidate parental samples), either using or not using a sibship size prior = 1. For all these analyses, we used custom‐generated R (R Development Core Team 2009) scripts (see Appendix S1) to run colony for multiple input files (settings: “medium” run length, “high” precision, 10 replicates for each analysis in each species), record estimates of *N*_b_, and calculate the average number of different mates per inferred breeder of each sex (a measure of the degree of polygamy) from inferred families. In all analyses using a sibship size prior, we set a “weak” prior in order to aid but not force family reconstruction.

## 3 RESULTS

### 3.1 CMR estimates of *N*_a_

**Figure 1:**
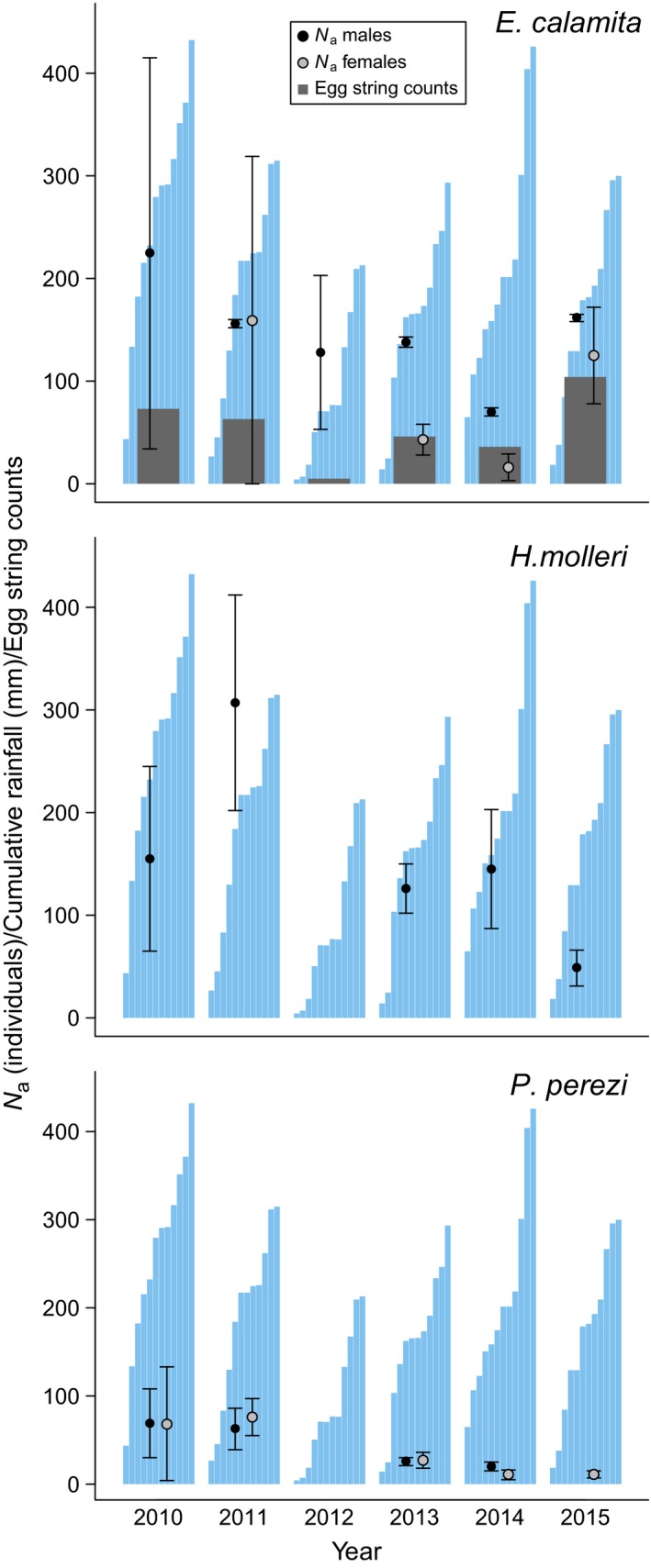
Annual estimates of *N*_a_ (with 95% CI) obtained in *Laguna de Valdemanco* for the three species, by sex (males: black circles; females: gray circles). Some *N*_a_ values could not be estimated (see Appendix S2). Dark gray bars show annual counts of egg strings of *E. calamita*. Blue bars show monthly cumulative rainfall data from the Barajas weather station (Madrid, about 40 km south from *Laguna de Valdemanco*). A sharp decrease in precipitation is apparent in 2012, especially in the early months of the year, when the breeding activity of the three species is concentrated

For each species, the top three ranked models encompassed more than 99% of the weight based on AICc scores (see Appendix S2). We did not detect consistent departures from CJS model assumptions among secondary occasions in any of the species, although males of *E. calamita* showed evidence of “transience” effect in 2011 (Table S2.2 in Appendix S2). Estimates of *N*_a_ were concordant in most years across different models (Appendix S2). Values obtained after weighted averaging across candidate models are shown in Table 1 (for the year of the tadpole genetic sampling in each species) and Figure 1. Estimated numbers of males of *E. calamita* and *H. molleri* were similar, around 150 individuals every year, although extreme high and low estimates were also obtained in some years (Figure 1). Unfortunately, the number of females of *H. molleri* could not be estimated due to their low recapture rate (0.04). Estimates of *N*_a_ in *E. calamita* and *H. molleri* clearly outnumbered those in *P. perezi*. The sex ratio of *P. perezi* in 2010 was very close to 1:1, and in the case of *E. calamita,* it was male‐biased in most years, especially in 2013 (Figure 1). Precision of *N*_a_ estimates, based on 95% CIs, improved with cumulative data from successive years in all three species, but especially in males of *E. calamita*, for which highly precise estimates were obtained from 2013 to 2015. For *H. molleri* and *P. perezi*, population declines became apparent in the period from 2012 to 2015. The year 2012 was unusually dry in *Laguna de Valdemanco*, as reflected in a sharp drop in egg string counts of *E. calamita* (from an average of 60 to only five egg strings, Figure 1).

### 3.2 Genetic estimates of *N*_b_

**Figure 2:**
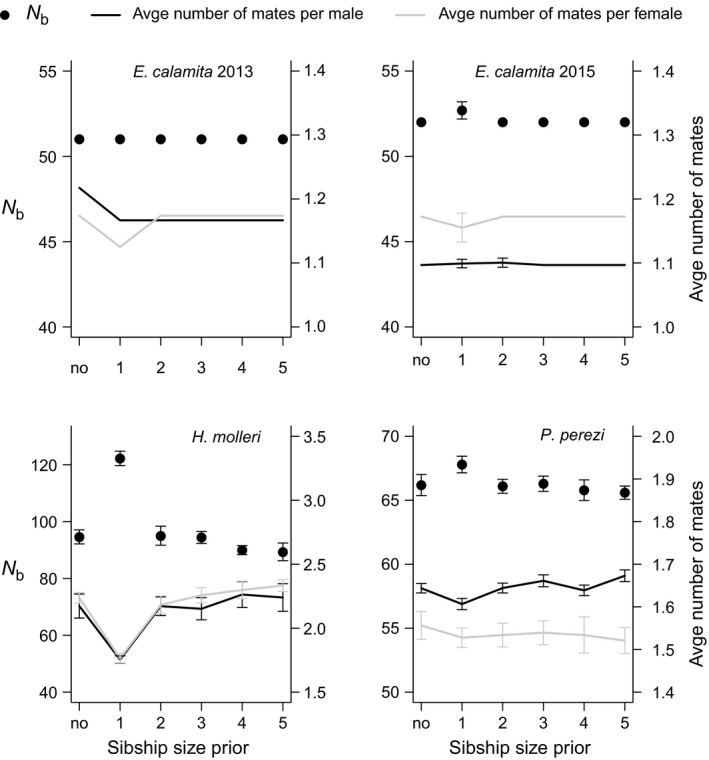
Estimates of *N*_b_ (dots) and average number of mates per breeder male (black lines) and female (gray lines) using different sibship size prior values (from one to five) or no prior (no). Estimates are averaged among ten replicates for each prior value (*N*_b_: harmonic mean; average number of mates: arithmetic mean). Error bars represent 95% CIs. Note that in the case of *E. calamita*, no variance among replicates was observed in all estimates in 2013 and in most estimates in 2015

**Figure 3:**
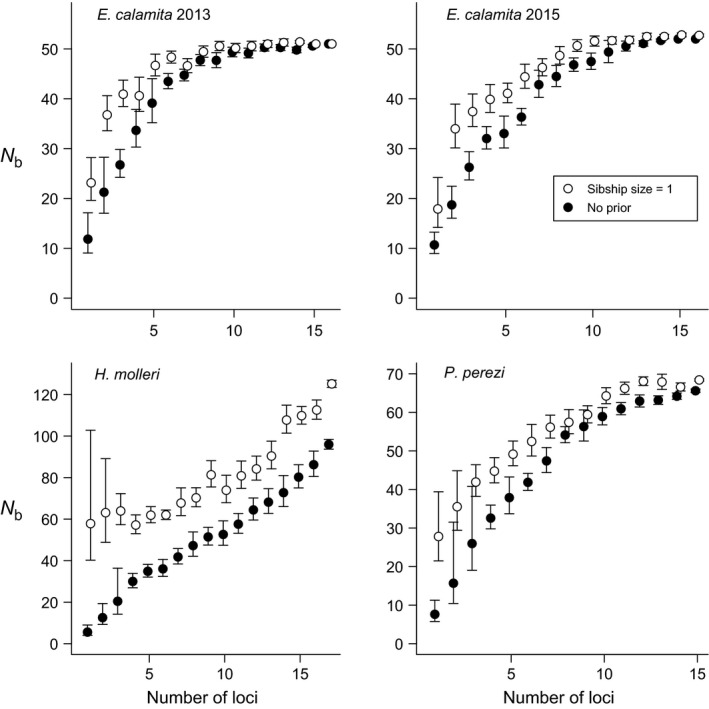
Harmonic means (with 95% CIs) of point estimates of *N*_b_ obtained in 10 replicated SF analyses using a sibship size prior = 1 (white dots) or no prior (black dots) with increasing marker information

**Figure 4:**
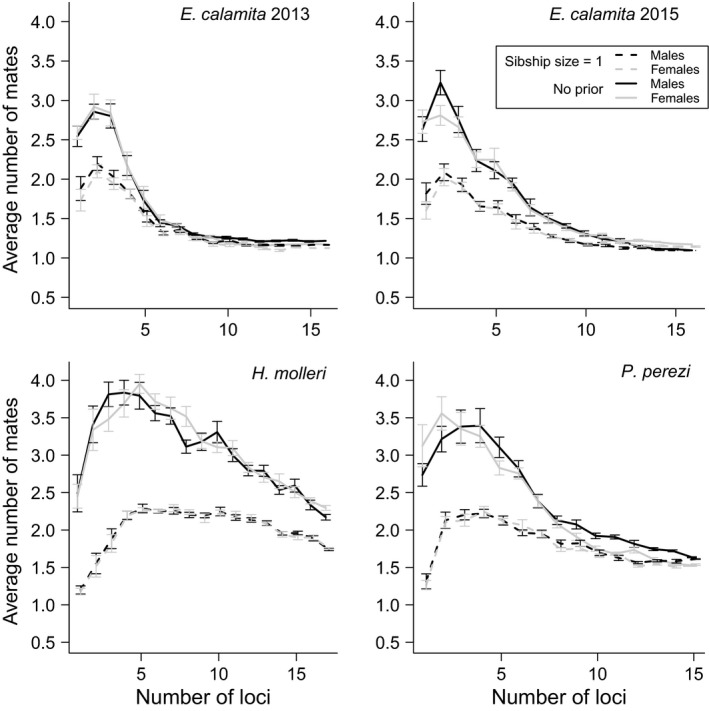
Arithmetic means (with 95% CIs) of the average number of mates per breeder male (dark lines) and female (gray lines) obtained in ten replicated SF analyses using a sibship size prior = 1 (dashed lines) or no prior (solid lines) with increasing marker information

**Figure 5:**
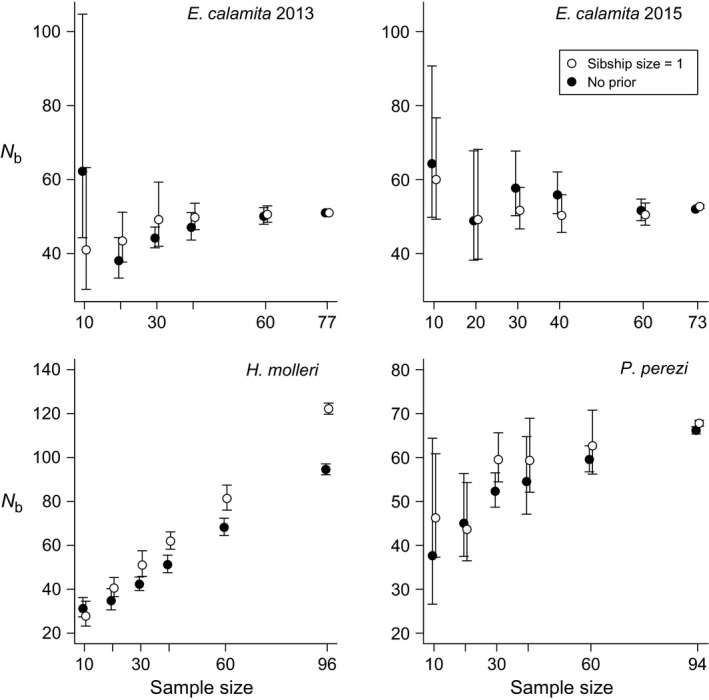
Harmonic means (with 95% CIs) of point estimates of *N*_b_ obtained in ten replicated SF analyses with different subsample sizes using a sibship size prior = 1 (white dots) or no prior (black dots)

**Figure 6:**
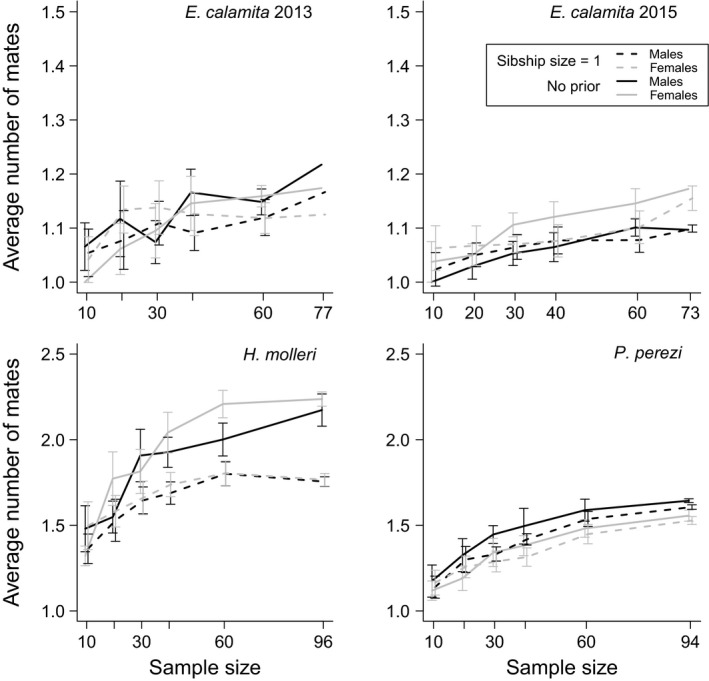
Arithmetic means (with 95% CIs) of the average number of mates per breeding male (dark lines) and female (gray lines) obtained with different subsample sizes in ten replicated SF analyses using a sibship size prior = 1 (dashed lines) or no prior (solid lines). Note the difference in axes scales

Estimates of *N*_b_ for *E. calamita* were slightly over 50 in both years, 2013 and 2015 (Table 1). For *H. molleri* and *P. perezi, N*_b_ estimates were 131 and 69, respectively (Table 1). These values, obtained with very long runs of the full datasets, were concordant with those obtained with medium length runs in the replicated analyses in the case of *E. calamita* and *P. perezi*, but not in *H. molleri* (as shown by the comparison of *N*_b_ values in Table 1 with final *N*_b_ values in Figures 2, 3, and 5). Between 46 and 87% of the inferred parents in reconstructed families of *E. calamita* and *P. perezi* were among the genotyped candidate fathers and mothers, but only 35% of the inferred sires and 10% of the inferred dams of *H. molleri* were included in the candidate parental samples (Table 1). These values were not affected by the use of a different prior probability for a true parent being included in the genotyped candidates (results not shown). The estimated average sibship sizes (and ranges) were 3.35 (1–6) for both sexes of *E. calamita* in 2013, 2.35 (1–8) and 2.52 (1–7) for paternal (p) and maternal (m) sibship sizes of *E. calamita* in 2015, 1.85 (1–5, p) and 1.88 (1–5, m) for *H. molleri,* and 2.54 (1–7, p) and 2.47 (1–9, m) for *P. perezi* (Appendix S3). We found low levels of polygamy in *E. calamita* both in 2013 and 2015 (Figures 2, 4 and 6). According to inferred parentage relationships, 83–90% of the successfully breeding males and 86–87% of the successfully breeding females of *E. calamita* mated with only one partner in each breeding season (Appendix S3). In contrast, higher polygamy levels were inferred in *H. molleri* (50% of inferred sires and 49% of inferred dams were polygamous) and *P. perezi* (46% of inferred sires and 42% of inferred dams were polygamous, see Figures 2, 4, and 6 and Appendix S3).

### 3.3 Sensitivity of *N*_b_ estimates to sibship size prior, sample size, and number of loci

Parentage assignment errors are possible due to limited parental sampling and/or insufficient marker information. For this reason, checking for the convergence of results with different analytical settings and different amounts of marker information is critical to assess the reliability of estimates. Using paternal and maternal sibship size priors = 1 resulted in an increase in *N*_b_ estimates and a proportional decrease in the average inferred number of mates per breeder in the three species (Figure 2). Using prior sibship size values between two and five yielded similar results to using no sibship size prior (Figure 2). Similar patterns were observed when comparing *N*_b_ and polygamy rates either using a sibship size prior = 1 or no prior at increasing levels of marker information (Figures 3 and 4): the use of the prior reduced inferred polygamy levels and increased *N*_b_ estimates in the three species. In the case of *E. calamita* and *P. perezi*, using the prior raised estimates of *N*_b_ when little marker information was provided (less than eight markers), thus approaching the convergent final estimates obtained with the full marker set. However, estimates in analyses with and without the sibship size prior in *H. molleri* did not reach convergence (Figures 3 and 4). There was also a clear convergence of estimates of *N*_b_ and (to a lesser extent) polygamy levels in *E. calamita* and *P. perezi* with increasing sample size (Figures 5 and 6). At small sample sizes (less than 30 larval genotypes, Figure 5), *N*_b_ estimates remained stable in *E. calamita*, but decreased in *P. perezi*. Results in *H. molleri* were, again, increasingly divergent with increasing sample size.

## 4 DISCUSSION

Estimation of the *N*_b_/*N*_a_ ratio is critically dependent on the accuracy of estimates of both *N*_b_ and *N*_a_. Independent field‐based information and replicated analyses play an invaluable role in assessing the reliability of results. In the case of *E. calamita,* we obtained *N*_b_/*N*_a_ ratios of 0.28 and 0.18 in 2013 and 2015, respectively (Table 1). These values are higher than the effective/census size ratios reported by Rowe and Beebee (2004, they calculated *N*_e_ rather than *N*_b_) and Beebee (2009) for similar census‐sized British populations (i.e., with *N*_a_ = 100–300), although both studies reported even higher ratios for small populations (>0.5 and 1, respectively). However, these two studies did not estimate *N*_a_ by CMR methods, but instead estimated the total number of breeding adults from counts of egg strings, on the basis that females of *E. calamita* usually lay a single egg string per year (Denton & Beebee, 1993). In our population, counts of egg strings of *E. calamita* provided a minimum estimate for the number of successfully mating females in the years of tadpole sampling (46 in 2013 and 104 in 2015) that very closely matched the number of potential breeding females estimated by the CMR method (43 in 2013 and 125 in 2015, see Table 1). These results are concordant with a female mating success close to one in our population, and support the hypothesis that counts of egg strings are a good surrogate for the number of breeding females. On the other hand, the estimated number of females and egg string counts clearly outnumbered the actual number of dams inferred in SF analyses (23 in 2013 and 29 in 2015, Table 1 and Appendix S3). This indicates that our offspring samples did not include a comprehensive representation of all the mating pairs of the year, likely due to high variance in reproductive success after accounting for offspring viability and survival in their initial developmental stages. This could be associated to the high mortality rate observed at the egg stage, due to early desiccation of the ephemeral pools and shores selected for breeding (we estimated a minimum of 16% of egg strings lost due to early pond desiccation in our study area, unpublished obs.). The high risk of breeding failure in *E. calamita* could result in differences in *N*_b_ depending on the sampling stage (e.g., eggs or metamorphic individuals), in contrast to species with preference for more predictable breeding sites (Phillipsen, Bowerman, & Blouin, 2010).

Effective/census size ratios in ranid frogs are typically higher than those reported for bufonid species (Hoffman, Schueler, & Blouin, 2004; Schmeller & Merilä, 2007). In our *P. perezi* population, we obtained an *N*_b_/*N*_a_ ratio of 0.5 (Table 1). This value is within the range reported for other ranid frogs (Brede & Beebee, 2006; Ficetola, Padoa‐Schioppa, Wang, & Garner, 2010; Phillipsen et al., 2010; Schmeller & Merilä, 2007), although this is the first study integrating both SF estimates of *N*_b_ and CMR estimates of *N*_a_. In *H. molleri*, only the number of adult males (126) could be estimated (Table 1, Figure 1), so we could not calculate the *N*_b_/*N*_a_ ratio in this species. Our sampling design does not seem to be optimally suited to provide reliable estimates of the number of adult females in *H. molleri* (see also Broquet et al., 2009; Pellet, Helfer, & Yannic, 2007). A specific CMR sampling scheme suited to the elusive breeding behavior of females of *H. molleri* should be adopted in the future to increase recapture rates.

### 4.1 Mating system inferences

The accuracy of *N*_b_ estimates obtained by SF analyses depends on the correct reconstruction of families, which can be hindered when genetic information is scarce or the sample size is small compared to the real (unknown) *N*_b_ of the population (Wang, 2016). Analyses of such datasets usually lead to unreliable family reconstruction mainly due to type I error inflation caused by misidentification of unrelated or loosely related (e.g., cousins) individuals as full or half sibs (Wang, 2016). In fact, false half‐sib assignations are far more common than false full‐sib identifications in cases of low marker information (Ackerman et al., 2017). This leads to inflated levels of polygamy and biased *N*_b_ estimates. For this reason, both exploration of inferred families and comparison with field observations of breeding activity are crucial cross‐check points to identify possible analytical artifacts.

In our study area, inferred levels of polygamy varied among different species. Oviposition in these three anuran species usually takes place when the female is in amplexus with only one male (Arak, 1988; García‐París et al., 2004; Lengagne & Joly, 2010). This suggests that each egg mass/string is only sired by one male and one female, but there is no empirical evidence for this, and thus, this question should be further addressed with the help of markers such as the microsatellites used here. During our 6‐year monitoring program, we detected individual males and females of *H. molleri* and *P. perezi* and males of *E. calamita* that remained in the breeding site during more than 30 days in a single breeding season, thereby providing some chances for multiple mating (Byrne & Roberts, 2012). In contrast, we only detected two females of *E. calamita* which remained more than 8 days in *Laguna de Valdemanco* in a single breeding season (11 and 15 days, respectively). Accordingly, we found low levels of female (but also male) polygamy in the reconstructed families of *E. calamita*, both in 2013 and 2015 (Figures 2, 4, and 6). The average full sibship size in reconstructed families of *E. calamita* was higher than 2.3 in both years, and most inferred parents were identified among the genotyped adults (Table 1 and Appendix S3). These results were independent of the use of any sibship size prior, thus supporting the reliability of mating system inferences. *Epidalea calamita* lays clutches in ephemeral puddles (therefore reducing interspecific but increasing intraspecific competition), taking advantage of their fast larval development (Gómez‐Mestre & Tejedo, 2002). Immediate occupancy of these ephemeral sites after heavy rainfalls is therefore critical for maximizing the opportunities for larvae to survive until metamorphosis. Within‐year monogamy might be a consequence of this breeding behavior.

In contrast, we obtained higher polygamy rates in *P. perezi* and *H. molleri* (Figures 2, 4, and 6 and Appendix S3), which is concordant with the longer time spent at the breeding site by individuals of both sexes in these species. In view of the relatively large *N*_b_/*N*_a_ ratio observed in *P. perezi* (Table 1), polyandry could be interpreted as a strategy that allows this species to maintain relatively high levels of genetic diversity in scenarios of low abundance (Byrne & Roberts, 2012; Lengagne & Joly, 2010). Similar genetic compensation effects have been previously documented in other anuran species (Beebee, 2009; Hinkson & Richter, 2016). Alternatively, polyandry in *P. perezi* could be a consequence of the long breeding period of this species, potentially associated to a risk‐spreading strategy involving spatial and temporal division of clutches (Byrne & Roberts, 2012). Contrasting *N*_b_/*N*_a_ ratios and levels of polygamy found in *E. calamita* and *P. perezi* suggests that amphibian species with different life‐history traits and breeding behavior may show different strategies aimed at maintaining genetic diversity at a local scale. This would imply that variation in hydroperiod length could, through effects on mating systems, affect the ability of different amphibian species to maintain genetic diversity, an interesting working hypothesis that deserves further exploration.

### 4.2 Sensitivity of N_b_ estimates to sibship size prior, sample size, and number of loci

In cases of artificially inflated polygamy in family reconstructions, setting a sibship size prior = 1 could aid sibship reconstruction by preventing false sib assignments. In *E. calamita* and *P. perezi*, replicated analyses with different sibship size priors, number of markers, and sample sizes were highly convergent (Figures 3, 4, 5, 6), supporting the reliability of our results. Thus, it was possible to compare the final results (obtained with the complete dataset and full marker information) with estimates obtained with subsampled datasets. In both species, the use of the lowest sibship size prior (i.e., = 1) led to better *N*_b_ estimates (i.e., closer to final estimates) in cases of both small sample size and low marker information (Figures 3 and 5). In addition, in *H. molleri,* the use of a low sibship size prior also reduced polygamy levels and increased the inferred number of parents and the corresponding *N*_b_ estimate. However, the lack of final convergent results highlights the need of additional genetic information to obtain reliable estimates of *N*_b_ and also to assess the magnitude of the effect of using the sibship size prior in this species. Integrative studies addressing *N*_b_ estimation by the SF method in multiple species and complemented with simulation studies will help provide general guidelines for the use of sibship size priors in SF analyses.

### 4.3 Extension to *N*_e_/*N*_a_ estimation

We have focused on the genetic estimate of *N*_b_, a parameter that intuitively relates to the number of breeders of the season (Waples & Antao, 2014). Amphibians are typically iteroparous breeders, but different species show wide variation in their maximum life span (García‐París et al., 2004). Our most time‐distant recaptures so far are 2 years for *H. molleri*, 5 years for *P. perezi,* and 6 years for *E. calamita*. All these individuals were initially marked as sexually mature adults, so time‐distant recaptures are an underestimate of their actual life span (Banks, Beebee, & Denton, 1993; Docampo & Milagrosa‐Vega, 1991; Esteban, García‐París, & Castanet, 1996; Leskovar, Oromi, Sanuy, & Sinsch, 2006; Patón et al., 1991; Pellet, Maze, & Perrin, 2006; Pellet, Schmidt, Fivaz, Perrin, & Grossenbacher, 2006). The integration of age information (for instance, from skeletochronological studies, Esteban et al., 1996; Friedl & Klump, 1997; Leskovar et al., 2006; Sinsch, 2015) with SF analyses would allow calculation of key parameters, like generation length and age variation in breeding success (Wang et al., 2010). In consequence, the effective size in a generation (*N*_e_) could be estimated and compared with census size inferences based on *N*_a_ estimates (Waples, 2005; Waples et al., 2011). As *N*_a_ is estimated from captures of adult individuals in the breeding sites, the variation of *N*_a_ over time will be due to mortality/natality processes and to the variation in dispersal rates and attendance to breeding sites driven by internal (e.g., energetic state) and environmental (e.g., meteorological conditions) factors (Cayuela et al., 2014; Cayuela, Arsovski, et al. 2016; Muths et al., 2006, 2013).

The ratio *N*_e_/*N*_a_ is more informative about evolutionary processes affecting populations at larger temporal scales. Distinguishing between intrinsic reproductive features and adaptive demographic strategies will require further exploration of *N*_e_/*N*_a_ in a network of populations. The increasing accessibility to hundreds of species‐specific molecular markers and the analytical versatility of SF analyses in colony for multiple species and mating systems, coupled with unparalleled computation power, provides great opportunities for integrative demographic research. This information will be in turn cornerstone for the interpretation of patterns of genetic structure at larger scales and thus for the implementation of effective conservation policies.

## DATA ACCESSIBILITY

The dryad archive (https://doi.org/10.5061/dryad.2fr3k)contains new microsatellite genotype data of the three species and the CMR capture histories.

## AUTHORS’ CONTRIBUTIONS

G.S.‐M. and I.M.‐S. designed the research and conducted fieldwork and sample collection. G.S.‐M. conducted laboratory work. G.S.‐M., J.W., J.L.V., and A.H.A. performed the genetic analyses. G.S.‐M., I.M.‐S. and J.W. led the writing of the manuscript. All authors contributed critically to the drafts and gave final approval for publication.

## CONFLICT OF INTEREST

None declared.

## References

[ece33387-bib-0001] Ackerman, M. W. , Hand, B. K. , Waples, R. K. , Luikart, G. , Waples, R. S. , Steele, C. A. , … Campbell, M. R. (2017). Effective number of breeders from sibship reconstruction: Empirical evaluations using hatchery steelhead. Evolutionary Applications, 10, 146–160. 28127391 10.1111/eva.12433 PMC5253425

[ece33387-bib-0002] Akaike, H. (1974). A new look at the statistical model identification. IEEE Transactions on Automatic Control, 19, 716–723.

[ece33387-bib-0003] Arak, A. (1988). Female mate selection in the natterjack toad: Active choice or passive attraction? Behavioral Ecology and Sociobiology, 22, 317–327.

[ece33387-bib-0004] Banks, B. , Beebee, T. J. C. , & Denton, J. S. (1993). Long‐term management of a natterjack toad (*Bufo calamita*) population in southern Britain. Amphibia‐Reptilia, 14, 155–168.

[ece33387-bib-0005] Banks, S. C. , Cary, G. J. , Smith, A. L. , Davies, I. D. , Driscoll, D. A. , Gill, A. M. , … Peakall, R. (2013). How does ecological disturbance influence genetic diversity? Trends in Ecology and Evolution, 28, 670–679. 24054910 10.1016/j.tree.2013.08.005

[ece33387-bib-0006] Beebee, T. J. C. (2009). A comparison of single‐sample effective size estimators using empirical toad (*Bufo calamita*) population data: Genetic compensation and population size‐genetic diversity correlations. Molecular Ecology, 18, 4790–4797. 19863715 10.1111/j.1365-294X.2009.04398.x

[ece33387-bib-0007] Bernos, T. A. , & Fraser, D. J. (2016). Spatiotemporal relationship between adult census size and genetic population size across a wide population size gradient. Molecular Ecology, 25, 4472–4487. 27483203 10.1111/mec.13790

[ece33387-bib-0008] Brede, E. G. , & Beebee, T. J. C. (2006). Large variations in the ratio of effective breeding and census population sizes between two species of pond‐breeding anurans. Biological Journal of the Linnean Society, 89, 365–372.

[ece33387-bib-0009] Broquet, T. , Jaquiéry, J. , & Perrin, N. (2009). Opportunity for sexual selection and effective population size in the lek‐breeding European treefrog (*Hyla arborea*). Evolution, 63, 674–683. 19087183 10.1111/j.1558-5646.2008.00586.x

[ece33387-bib-0010] Burnham, K. P. , & Anderson, D. R. (2002). Model selection and multimodel inference: A practical information‐theoretic approach, 2nd ed. New York: Springer Science & Business Media.

[ece33387-bib-0011] Byrne, P. G. , & Roberts, J. D. (2012). Evolutionary causes and consequences of sequential polyandry in anuran amphibians. Biological Reviews, 87, 209–228. 21740503 10.1111/j.1469-185X.2011.00191.x

[ece33387-bib-0012] Caballero, A. (1994). Developments in the prediction of effective population size. Heredity, 73, 657–679. 7814264 10.1038/hdy.1994.174

[ece33387-bib-0013] Cadeddu, G. , & Castellano, S. (2012). Factors affecting variation in the reproductive investment of female treefrogs, *Hyla intermedia* . Zoology, 115, 372–378. 23022366 10.1016/j.zool.2012.04.006

[ece33387-bib-0014] Cayuela, H. , Arsovski, D. , Thirion, J.‐M. , Bonnaire, E. , Pichenot, J. , Boitaud, S. , … Besnard, A. (2016). Contrasting patterns of environmental fluctuation contribute to divergent life histories among amphibian populations. Ecology, 97, 980–991. 27220214

[ece33387-bib-0015] Cayuela, H. , Besnard, A. , Bonnaire, E. , Perret, H. , Rivoalen, J. , Miaud, C. , & Joly, P. (2014). To breed or not to breed: Past reproductive status and environmental cues drive current breeding decisions in a long‐lived amphibian. Oecologia, 176, 107–116. 24996543 10.1007/s00442-014-3003-x

[ece33387-bib-0016] Cayuela, H. , Boualit, L. , Arsovski, D. , Bonnaire, E. , Pichenot, J. , Bellec, A. , … Besnard, A. (2016). Does habitat unpredictability promote the evolution of a colonizer syndrome in amphibian metapopulations? Ecology, 97, 2658–2670. 27859109 10.1002/ecy.1489

[ece33387-bib-0017] Charlesworth, B. (2009). Effective population size and patterns of molecular evolution and variation. Nature Reviews Genetics, 10, 195–205. 10.1038/nrg2526 19204717

[ece33387-bib-0018] Choquet, R. , Lebreton, J.‐D. , Gimenez, O. , Reboulet, A.‐M. , & Pradel, R. (2009). U‐CARE: Utilities for performing goodness of fit tests and manipulating CApture‐REcapture data. Ecography, 32, 1071–1074.

[ece33387-bib-0019] Clutton‐Brock, T. , & Sheldon, B. C. (2010). Individuals and populations: The role of long‐term, individual‐based studies of animals in ecology and evolutionary biology. Trends in Ecology and Evolution, 25, 562–573. 20828863 10.1016/j.tree.2010.08.002

[ece33387-bib-0020] Crow, J. F. , & Kimura, M. (1970). An introduction to population genetics theory. Caldwell, NJ: The Blackburn Press.

[ece33387-bib-0021] Denton, J. S. , & Beebee, T. J. C. (1993). Density‐related features of natterjack toad (*Bufo calamita*) populations in Britain. Journal of Zoology, 229, 105–119.

[ece33387-bib-0022] Denton, J. S. , & Beebee, T. J. C. (1996). Double‐clutching by natterjack toads *Bufo calamita* at a site in Southern England. Amphibia‐Reptilia, 17, 159–167.

[ece33387-bib-0023] Docampo, L. , & Milagrosa‐Vega, M. (1991). Determinación de la edad en *Rana perezi* Seoane, 1885. Aplicación al análisis del crecimiento somático de poblaciones. Doñana, Acta Vertebrata, 18, 21–38.

[ece33387-bib-0024] Esteban, M. , García‐París, M. , & Castanet, J. (1996). Use of bone histology in estimating the age of frogs (*Rana perezi*) from a warm temperate climate area. Canadian Journal of Zoology, 74, 1914–1921.

[ece33387-bib-0025] Ficetola, G. F. , Padoa‐Schioppa, E. , Wang, J. , & Garner, T. W. J. (2010). Polygyny, census and effective population size in the threatened frog, *Rana latastei* . Animal Conservation, 13, 82–89.

[ece33387-bib-0026] Frankham, R. (1995). Effective population size/adult population size ratios in wildlife: A review. Genetical Research, 66, 95–107. 10.1017/S0016672308009695 18976539

[ece33387-bib-0027] Fraser, D. J. , Hansen, M. M. , Østergaard, S. , Tessier, N. , Legault, M. , & Bernatchez, L. (2007). Comparative estimation of effective population sizes and temporal gene flow in two contrasting population systems. Molecular Ecology, 16, 3866–3889. 17850551 10.1111/j.1365-294X.2007.03453.x

[ece33387-bib-0028] Friedl, T. W. P. , & Klump, G. M. (1997). Some aspects of population biology in the European treefrog, *Hyla arborea* . Herpetologica, 53, 321–330.

[ece33387-bib-0029] García‐París, M. , Montori, A. , & Herrero, P. (2004). Amphibia, Lissamphibia In Ramos M. A., Alba J., Bellés i Ros X., Gosálbez i Noguera J., Guerra A., Macpherson E., Serrano J. & Templado J. (Eds.), Fauna ibérica (pp. 640). Madrid: Museo Nacional de Ciencias Naturales–CSIC.

[ece33387-bib-0030] Gómez‐Mestre, I. , & Tejedo, M. (2002). Geographic variation in asymmetric competition: A case study with two larval anuran species. Ecology, 83, 2102–2111.

[ece33387-bib-0031] Gomez‐Uchida, D. , Palstra, F. P. , Knight, T. W. , & Ruzzante, D. E. (2013). Contemporary effective population and metapopulation size (*N* _*e*_ and meta‐*N* _*e*_): Comparison among three salmonids inhabiting a fragmented system and differing in gene flow and its asymmetries. Ecology and Evolution, 3, 569–580. 23532448 10.1002/ece3.485 PMC3605847

[ece33387-bib-0032] Hinkson, K. M. , & Richter, S. C. (2016). Temporal trends in genetic data and effective population size support efficacy of management practices in critically endangered dusky gopher frogs (*Lithobates sevosus*). Ecology and Evolution, 6, 2667–2678. 27066242 10.1002/ece3.2084 PMC4798149

[ece33387-bib-0033] Hoffman, E. A. , Schueler, F. W. , & Blouin, M. S. (2004). Effective population sizes and temporal stability of genetic structure in *Rana pipiens*, the northern leopard frog. Evolution, 58, 2536–2545. 15612296 10.1111/j.0014-3820.2004.tb00882.x

[ece33387-bib-0034] Jones, O. R. , & Wang, J. (2010). colony: A program for parentage and sibship inference from multilocus genotype data. Molecular Ecology Resources, 10, 551–555. 21565056 10.1111/j.1755-0998.2009.02787.x

[ece33387-bib-0035] Kamath, P. L. , Haroldson, M. A. , Luikart, G. , Paetkau, D. , Whitman, C. , & Van Manen, F. T. (2015). Multiple estimates of effective population size for monitoring a long‐lived vertebrate: An application to Yellowstone grizzly bears. Molecular Ecology, 24, 5507–5521. 26510936 10.1111/mec.13398

[ece33387-bib-0036] Kendall, W. L. , & Nichols, J. D. (1995). On the use of secondary capture‐recapture samples to estimate temporary emigration and breeding proportions. Journal of Applied Statistics, 22, 751–762.

[ece33387-bib-0037] Kendall, W. L. , Nichols, J. D. , & Hines, J. E. (1997). Estimating temporary emigration using capture‐recapture data with Pollock's robust design. Ecology, 78, 563–578.

[ece33387-bib-0038] Kendall, W. L. , Pollock, K. H. , & Brownie, C. (1995). A likelihood‐based approach to capture‐recapture estimation of demographic parameters under the robust design. Biometrics, 51, 293–308. 7766783

[ece33387-bib-0039] Lebreton, J.‐D. , Burnham, K. P. , Clobert, J. , & Anderson, D. R. (1992). Modeling survival and testing biological hypotheses using marked animals: A unified approach with case studies. Ecological Monographs, 62, 67–118.

[ece33387-bib-0040] Lengagne, T. , & Joly, P. (2010). Paternity control for externally fertilised eggs: Behavioural mechanisms in the waterfrog species complex. Behavioral Ecology and Sociobiology, 64, 1179–1186.

[ece33387-bib-0041] Leskovar, C. , Oromi, N. , Sanuy, D. , & Sinsch, U. (2006). Demographic life history traits of reproductive natterjack toads (*Bufo calamita*) vary between northern and southern latitudes. Amphibia‐Reptilia, 27, 365–375.

[ece33387-bib-0042] Luikart, G. , Ryman, N. , Tallmon, D. A. , Schwartz, M. K. , & Allendorf, F. W. (2010). Estimation of census and effective population sizes: The increasing usefulness of DNA‐based approaches. Conservation Genetics, 11, 355–373.

[ece33387-bib-0043] Manier, M. K. , & Arnold, S. J. (2005). Population genetic analysis identifies source–sink dynamics for two sympatric garter snake species (*Thamnophis elegans* and *Thamnophis sirtalis*). Molecular Ecology, 14, 3965–3976. 16262852 10.1111/j.1365-294X.2005.02734.x

[ece33387-bib-0044] Manier, M. K. , & Arnold, S. J. (2006). Ecological correlates of population genetic structure: A comparative approach using a vertebrate metacommunity. Proceedings of the Royal Society B, 273, 3001–3009. 17015357 10.1098/rspb.2006.3678 PMC1639520

[ece33387-bib-0045] McCarthy, M. A. , & Parris, K. M. (2004). Clarifying the effect of toe clipping on frogs with Bayesian statistics. Journal of Applied Ecology, 41, 780–786.

[ece33387-bib-0046] Muths, E. , Scherer, R. D. , & Bosch, J. (2013). Evidence for plasticity in the frequency of skipped breeding opportunities in common toads. Population Ecology, 55, 535–544.

[ece33387-bib-0047] Muths, E. , Scherer, R. D. , Corn, P. S. , & Lambert, B. A. (2006). Estimation of temporary emigration in male toads. Ecology, 87, 1048–1056. 16676548 10.1890/0012-9658(2006)87[1048:eoteim]2.0.co;2

[ece33387-bib-0048] Nunney, L. (1993). The influence of mating system and overlapping generations on effective population size. Evolution, 47, 1329–1341. 28564896 10.1111/j.1558-5646.1993.tb02158.x

[ece33387-bib-0049] Palstra, F. P. , & Fraser, D. J. (2012). Effective/census population size ratio estimation: A compendium and appraisal. Ecology and Evolution, 2, 2357–2365. 23139893 10.1002/ece3.329 PMC3488685

[ece33387-bib-0050] Palstra, F. P. , & Ruzzante, D. E. (2008). Genetic estimates of contemporary effective population size: What can they tell us about the importance of genetic stochasticity for wild population persistence? Molecular Ecology, 17, 3428–3447. 19160474 10.1111/j.1365-294x.2008.03842.x

[ece33387-bib-0051] Patón, D. , Juarranz, A. , Sequeros, E. , Pérez‐Campo, R. , López‐Torres, M. , & Barja de Quiroga, G. (1991). Seasonal age and sex structure of *Rana perezi* assessed by skeletochronology. Journal of Herpetology, 25, 389–394.

[ece33387-bib-0052] Pellet, J. , Helfer, V. , & Yannic, G. (2007). Estimating population size in the European tree frog (*Hyla arborea*) using individual recognition and chorus counts. Amphibia‐Reptilia, 28, 287–294.

[ece33387-bib-0053] Pellet, J. , Maze, G. , & Perrin, N. (2006). The contribution of patch topology and demographic parameters to population viability analysis predictions: The case of the European tree frog. Population Ecology, 48, 353–361.

[ece33387-bib-0054] Pellet, J. , Schmidt, B. R. , Fivaz, F. , Perrin, N. , & Grossenbacher, K. (2006). Density, climate and varying return points: An analysis of long‐term population fluctuations in the threatened European tree frog. Oecologia, 149, 65–71. 16779559 10.1007/s00442-006-0432-1

[ece33387-bib-0055] Phillipsen, I. C. , Bowerman, J. , & Blouin, M. (2010). Effective number of breeding adults in Oregon spotted frogs (*Rana pretiosa*): Genetic estimates at two life stages. Conservation Genetics, 11, 737–745.

[ece33387-bib-0056] Pollock, K. H. (1982). A capture‐recapture design robust to unequal probability of capture. Journal of Wildlife Management, 46, 752–757.

[ece33387-bib-0057] R Development Core Team (2009). R: A language and environment for statistical computing. Vienna, Austria: R Foundation for Statistical Computing.

[ece33387-bib-0058] Rowe, G. , & Beebee, T. J. C. (2004). Reconciling genetic and demographic estimators of effective population size in the anuran amphibian *Bufo calamita* . Conservation Genetics, 5, 287–298.

[ece33387-bib-0059] Ruzzante, D. E. , McCracken, G. R. , Parmelee, S. , Hill, K. , Corrigan, A. , MacMillan, J. , & Walde, S. J. (2016). Effective number of breeders, effective population size and their relationship with census size in an iteroparous species, *Salvelinus fontinalis* . Proceedings of the Royal Society B, 283, 20152601. 26817773 10.1098/rspb.2015.2601 PMC4795031

[ece33387-bib-0060] Sánchez‐Montes, G. , Ariño, A. H. , Vizmanos, J. L. , Wang, J. , & Martínez‐Solano, I. (2017). Effects of sample size and full sibs on genetic diversity characterization: A case study of three syntopic Iberian pond‐breeding amphibians. Journal of Heredity, 108, 535–543. 28444211 10.1093/jhered/esx038

[ece33387-bib-0061] Sánchez‐Montes, G. , & Martínez‐Solano, I. (2011). Population size, habitat use and movement patterns during the breeding season in a population of Perez's frog (*Pelophylax perezi*) in central Spain. Basic and Applied Herpetology, 25, 81–96.

[ece33387-bib-0062] Sánchez‐Montes, G. , Recuero, E. , Gutiérrez‐Rodríguez, J. , Gomez‐Mestre, I. , & Martínez‐Solano, I. (2016). Species assignment in the *Pelophylax ridibundus* x *P. perezi* hybridogenetic complex based on 16 newly characterised microsatellite markers. Herpetological Journal, 26, 99–108.

[ece33387-bib-0063] Schmeller, D. S. , & Merilä, J. (2007). Demographic and genetic estimates of effective population and breeding size in the amphibian *Rana temporaria* . Conservation Biology, 21, 142–151. 17298520 10.1111/j.1523-1739.2006.00554.x

[ece33387-bib-0064] Schwartz, M. K. , Tallmon, D. A. , & Luikart, G. (1998). Review of DNA‐based census and effective population size estimators. Animal Conservation, 1, 293–299.

[ece33387-bib-0065] Schwarz, C. J. , & Stobo, W. T. (1997). Estimating temporary migration using the robust design. Biometrics, 53, 178–194.

[ece33387-bib-0066] Silverin, B. , & Andrén, C. (1992). The ovarian cycle in the natterjack, *Bufo calamita*, and its relation to breeding behaviour. Amphibia‐Reptilia, 13, 177–192.

[ece33387-bib-0067] Sinsch, U. (2015). Review: Skeletochronological assessment of demographic life‐history traits in amphibians. Herpetological Journal, 25, 5–13.

[ece33387-bib-0068] Tavecchia, G. , Besbeas, P. , Coulson, T. , Morgan, B. J. T. , & Clutton‐Brock, T. H. (2009). Estimating population size and hidden demographic parameters with state‐space modeling. The American Naturalist, 173, 722–733. 10.1086/598499 19355815

[ece33387-bib-0069] Vucetich, J. A. , & Waite, T. A. (1998). Number of censuses required for demographic estimation of effective population size. Conservation Biology, 12, 1023–1030.

[ece33387-bib-0070] Wang, J. (2005). Estimation of effective population sizes from data on genetic markers. Philosophical Transactions of the Royal Society B, 360, 1395–1409. 10.1098/rstb.2005.1682 PMC1847562 16048783

[ece33387-bib-0071] Wang, J. (2009). A new method for estimating effective population sizes from a single sample of multilocus genotypes. Molecular Ecology, 18, 2148–2164. 19389175 10.1111/j.1365-294X.2009.04175.x

[ece33387-bib-0072] Wang, J. (2016). A comparison of single‐sample estimators of effective population sizes from genetic marker data. Molecular Ecology, 25, 4692–4711. 27288989 10.1111/mec.13725

[ece33387-bib-0073] Wang, J. , Brekke, P. , Huchard, E. , Knapp, L. A. , & Cowlishaw, G. (2010). Estimation of parameters of inbreeding and genetic drift in populations with overlapping generations. Evolution, 64, 1704–1718. 20100220 10.1111/j.1558-5646.2010.00953.x

[ece33387-bib-0074] Wang, J. , Santiago, E. , & Caballero, A. (2016). Prediction and estimation of effective population size. Heredity, 117, 193–206. 27353047 10.1038/hdy.2016.43 PMC5026755

[ece33387-bib-0075] Waples, R. S. (2005). Genetic estimates of contemporary effective population size: To what time periods do the estimates apply? Molecular Ecology, 14, 3335–3352. 16156807 10.1111/j.1365-294X.2005.02673.x

[ece33387-bib-0076] Waples, R. S. (2016). Life‐history traits and effective population size in species with overlapping generations revisited: The importance of adult mortality. Heredity, 117, 241–250. 27273324 10.1038/hdy.2016.29 PMC5026752

[ece33387-bib-0077] Waples, R. S. , & Antao, T. (2014). Intermittent breeding and constraints on litter size: Consequences for effective population size per generation (*N* _*e*_) and per reproductive cycle (*N* _*b*_). Evolution, 68, 1722–1734. 24611912 10.1111/evo.12384

[ece33387-bib-0078] Waples, R. S. , Antao, T. , & Luikart, G. (2014). Effects of overlapping generations on linkage disequilibrium estimates of effective population size. Genetics, 197, 769–780. 24717176 10.1534/genetics.114.164822 PMC4063931

[ece33387-bib-0079] Waples, R. S. , Do, C. , & Chopelet, J. (2011). Calculating *N* _*e*_ and *N* _*e*_ */N* in age‐structured populations: A hybrid Felsenstein‐Hill approach. Ecology, 92, 1513–1522. 21870625 10.1890/10-1796.1

[ece33387-bib-0080] Waples, R. S. , Luikart, G. , Faulkner, J. R. , & Tallmon, D. A. (2013). Simple life‐history traits explain key effective population size ratios across diverse taxa. Proceedings of the Royal Society B, 280, 20131339. 23926150 10.1098/rspb.2013.1339 PMC3757969

[ece33387-bib-0081] White, G. C. , & Burnham, K. P. (1999). Program MARK: Survival estimation from populations of marked animals. Bird Study, 46, s120–s139.

[ece33387-bib-0082] Whiteley, A. R. , Coombs, J. A. , O'Donnell, M. J. , Nislow, K. H. , & Letcher, B. H. (2017). Keeping things local: Subpopulation *N* _*b*_ and *N* _*e*_ in a stream network with partial barriers to fish migration. Evolutionary Applications, 10, 348–365. 28352295 10.1111/eva.12454 PMC5367083

[ece33387-bib-0083] Wright, S. (1931). Evolution in Mendelian populations. Genetics, 16, 97–159. 17246615 10.1093/genetics/16.2.97 PMC1201091

