# Supplementary material for: Reliable effective number of breeders/adult census size ratios in seasonal‐breeding species: Opportunity for integrative demographic inferences based on capture–mark–recapture data and multilocus genotypes
Source: Ecol Evol. 2017 Oct 28;7(23):10301–14. doi: 10.1002/ece3.3387 (PMC5723585; doi:10.1002/ece3.3387)
Supplement: Supplementary file 1 [file ECE3-7-10301-s001.docx]

**Appendix S1.** R scripts for replicated analyses.

# ------------------------------------------------

# APPENDIX S1. R scripts for replicated analyses

# ------------------------------------------------

#

# R scripts employed for replicated analyses exploring different sibship size prior values and different amounts of marker information (i.e., subsampling the #number of markers or the sample size).

# The three scripts use the same input file (named ‘inputfile.csv’) which should have 'm' offspring, 'x' candidate fathers and 'y' candidate mothers genotyped at #'n' loci, and arranged in the following format:

#

# ID;sex_stage;Loc1;Loc1_b;Loc2;Loc2_b;Loc3;Loc3_b;...;Locn;Locn_b;

# offspring1_ID;offspring;Loc1(allele1);Loc1(allele2);Loc2(allele1);Loc2(allele2);Loc3(allele1);Loc3(allele2);...;Locn(allele1);Locn(allele2)

# offspring2_ID;offspring;Loc1(allele1);Loc1(allele2);Loc2(allele1);Loc2(allele2);Loc3(allele1);Loc3(allele2);...;Locn(allele1);Locn(allele2)

# offspring3_ID;offspring;Loc1(allele1);Loc1(allele2);Loc2(allele1);Loc2(allele2);Loc3(allele1);Loc3(allele2);...;Locn(allele1);Locn(allele2)

# offspring4_ID;offspring;Loc1(allele1);Loc1(allele2);Loc2(allele1);Loc2(allele2);Loc3(allele1);Loc3(allele2);...;Locn(allele1);Locn(allele2)

# offspring5_ID;offspring;Loc1(allele1);Loc1(allele2);Loc2(allele1);Loc2(allele2);Loc3(allele1);Loc3(allele2);...;Locn(allele1);Locn(allele2)

# offspring6_ID;offspring;Loc1(allele1);Loc1(allele2);Loc2(allele1);Loc2(allele2);Loc3(allele1);Loc3(allele2);...;Locn(allele1);Locn(allele2)

# ...

# offspringm_ID;offspring;Loc1(allele1);Loc1(allele2);Loc2(allele1);Loc2(allele2);Loc3(allele1);Loc3(allele2);...;Locn(allele1);Locn(allele2)

# male1_ID;male;Loc1(allele1);Loc1(allele2);Loc2(allele1);Loc2(allele2);Loc3(allele1);Loc3(allele2);...;Locn(allele1);Locn(allele2)

# male2_ID;male;Loc1(allele1);Loc1(allele2);Loc2(allele1);Loc2(allele2);Loc3(allele1);Loc3(allele2);...;Locn(allele1);Locn(allele2)

# male3_ID;male;Loc1(allele1);Loc1(allele2);Loc2(allele1);Loc2(allele2);Loc3(allele1);Loc3(allele2);...;Locn(allele1);Locn(allele2)

# male4_ID;male;Loc1(allele1);Loc1(allele2);Loc2(allele1);Loc2(allele2);Loc3(allele1);Loc3(allele2);...;Locn(allele1);Locn(allele2)

# ...

# malex_ID;male;Loc1(allele1);Loc1(allele2);Loc2(allele1);Loc2(allele2);Loc3(allele1);Loc3(allele2);...;Locn(allele1);Locn(allele2)

# female1_ID;male;Loc1(allele1);Loc1(allele2);Loc2(allele1);Loc2(allele2);Loc3(allele1);Loc3(allele2);...;Locn(allele1);Locn(allele2)

# female2_ID;male;Loc1(allele1);Loc1(allele2);Loc2(allele1);Loc2(allele2);Loc3(allele1);Loc3(allele2);...;Locn(allele1);Locn(allele2)

# female3_ID;male;Loc1(allele1);Loc1(allele2);Loc2(allele1);Loc2(allele2);Loc3(allele1);Loc3(allele2);...;Locn(allele1);Locn(allele2)

# female4_ID;male;Loc1(allele1);Loc1(allele2);Loc2(allele1);Loc2(allele2);Loc3(allele1);Loc3(allele2);...;Locn(allele1);Locn(allele2)

# ...

# femaley_ID;male;Loc1(allele1);Loc1(allele2);Loc2(allele1);Loc2(allele2);Loc3(allele1);Loc3(allele2);...;Locn(allele1);Locn(allele2)

#

#

#

# The input file and the executable file of program COLONY (Colony2P.exe) should be placed in the working directory.

# Notations in the right hand of the scripts (following a #) indicate that specific values and settings need to be included in the corresponding coding line, # following COLONY user guide.

# The specific settings included in these example scripts correspond to analyses of the E. calamita 2013 dataset of this paper.

#

#

# If you use the scripts for publishing papers, please cite Sánchez-Montes et al. 2017 Journal of Animal Ecology paper (Appendix S1).

#

#

#

#-----------------------------------------------

# Analyses for exploring different prior values

#-----------------------------------------------

inputfile<-read.csv(file="inputfile.csv", header=T, sep=";")

outputfile<-"outputfile.txt"

cat("filename","prior","totmal","totfem","Polymal","Polyfem","Avgemal","Avgefem","Ne","Ne_min","Ne_max",file=outputfile,append=TRUE,sep = ",",fill=TRUE)

offspring<-subset(inputfile, inputfile$sex_stage=="offspring")

offspring<-offspring[c(-2)]

cmales<-subset(inputfile, inputfile$sex_stage=="male")

cmales<-cmales[c(-2)]

cfemales<-subset(inputfile, inputfile$sex_stage=="female")

cfemales<-cfemales[c(-2)]

for (i in 1:5) { #Set the desired prior values to explore

for (j in 1:10) { #Set the desired number of replicates for each prior value

filename<-paste("analysis",i,"prior",j,sep="_")

input.file<-paste(filename,".dat", sep="")

options(width=1000)

cat(filename, file=input.file, append=TRUE, sep = "\n")

cat(filename, file=input.file, append=TRUE, sep = "\n")

cat(length(offspring$ID), file=input.file, append=TRUE, sep = "\n")

cat((ncol(offspring)-1)/2, file=input.file, append=TRUE, sep = "\n")

cat(sample(100000, 1), file=input.file, append=TRUE, sep = "\n")

cat(0, file=input.file, append=TRUE, sep = "\n") #0/1=Not updating/updating allele frequency

cat(2, file=input.file, append=TRUE, sep = "\n") #2/1=Dioecious/Monoecious species

cat(0, file=input.file, append=TRUE, sep = "\n") #0/1=Inbreeding absent/present

cat(0, file=input.file, append=TRUE, sep = "\n") #0/1=Diploid species/HaploDiploid species

cat("0 0", file=input.file, append=TRUE, sep = "\n") #0/1=Polygamy/Monogamy for males & females

cat(0, file=input.file, append=TRUE, sep = "\n") #0/1 = Clone inference = No/Yes

cat(0, file=input.file, append=TRUE, sep = "\n") #0/1=Scale full sibship=No/Yes

cat(paste(1, i, i, sep=" "), file=input.file, append=TRUE, sep = "\n") #0/1/2/3/4=No/Weak/Medium/Strong sibship prior; 4=Optimal sibship prior for Ne

cat(0, file=input.file, append=TRUE, sep = "\n") #0/1=Unknown/Known population allele frequency

cat(1, file=input.file, append=TRUE, sep = "\n") #Number of runs

cat(2, file=input.file, append=TRUE, sep = "\n") #1/2/3/4 = Short/Medium/Long/VeryLong run

cat(0, file=input.file, append=TRUE, sep = "\n") #0/1=Monitor method by Iterate#/Time in second

cat("100000", file=input.file, append=TRUE, sep = "\n") #Monitor interval in Iterate# / in seconds

cat(0, file=input.file, append=TRUE, sep = "\n") #0/1=DOS/Windows version

cat(1, file=input.file, append=TRUE, sep = "\n") #0/1/2=Pair-Likelihood-Score(PLS)/Full-Likelihood(FL)/FL-PLS-combined(FPLS) method

cat(1, file=input.file, append=TRUE, sep = "\n") #0/1/2/3=Low/Medium/High/VeryHigh precision

cat(" ", file=input.file, append=TRUE, sep = "\n")

cat(names(offspring)[seq(from=2, to=ncol(offspring), by=2)], file=input.file, append=TRUE, sep = ",", fill=TRUE)

cat(rep(0,ncol(offspring)/2), file=input.file, append=TRUE, sep = ",", fill=TRUE) #Marker types, 0/1=Codominant/Dominant

cat(rep(0.05,(ncol(offspring)-1)/2), file=input.file, append=TRUE, sep = ",", fill=TRUE) #Allelic dropout rate at each locus

cat(rep(0.05,(ncol(offspring)-1)/2), file=input.file, append=TRUE, sep = ",", fill=TRUE) #Other typing error rate at each locus

cat(" ", file=input.file, append=TRUE, sep = "\n")

for (k in 1:nrow(offspring)){

cat(as.matrix(offspring[k,]), file=input.file, append=TRUE, sep = " ", fill=TRUE)

}

cat(" ", file=input.file, append=TRUE, sep = "\n")

cat("0.66 0.61", file=input.file, append=TRUE, sep = " ", fill=TRUE) #probabilities that the father and mother of an offspring are included in candidates

cat(c(nrow(cmales),nrow(cfemales)), file=input.file, append=TRUE, sep = " ", fill=TRUE)

cat(" ", file=input.file, append=TRUE, sep = "\n")

for (l in 1:nrow(cmales)){

cat(as.matrix(cmales[l,]), file=input.file, append=TRUE, sep = " ", fill=TRUE)

}

cat(" ", file=input.file, append=TRUE, sep = "\n")

for (m in 1:nrow(cfemales)){

cat(as.matrix(cfemales[m,]), file=input.file, append=TRUE, sep = " ", fill=TRUE)

}

cat(" ", file=input.file, append=TRUE, sep = "\n")

cat(0, file=input.file, append=TRUE, sep = "\n") #Number of offspring with known paternity

#IDs of known offspring-father dyad (if any)

cat(" ", file=input.file, append=TRUE, sep = "\n")

cat(5, file=input.file, append=TRUE, sep = "\n") #Number of offspring with known maternity

cat("GSC081 BC09421", file=input.file, append=TRUE, sep = "\n") #IDs of known offspring-mother dyad (if any)

cat("GSC082 BC09421", file=input.file, append=TRUE, sep = "\n") #IDs of known offspring-mother dyad (if any)

cat("GSC083 BC09421", file=input.file, append=TRUE, sep = "\n") #IDs of known offspring-mother dyad (if any)

cat("GSC084 BC09421", file=input.file, append=TRUE, sep = "\n") #IDs of known offspring-mother dyad (if any)

cat("GSC085 BC09421", file=input.file, append=TRUE, sep = "\n") #IDs of known offspring-mother dyad (if any)

cat(" ", file=input.file, append=TRUE, sep = "\n")

cat(0, file=input.file, append=TRUE, sep = "\n") #Number of known paternal sibship

#Size of known paternal sibship, and IDs of offspring in the sibship (if any)

cat(" ", file=input.file, append=TRUE, sep = "\n")

cat(0, file=input.file, append=TRUE, sep = "\n") #Number of known maternal sibship

#Size of known maternal sibship, and IDs of offspring in the sibship (if any)

cat(" ", file=input.file, append=TRUE, sep = "\n")

cat(0, file=input.file, append=TRUE, sep = "\n") #Number of offspring with known excluded paternity

#Offspring ID, number of excluded males, the IDs of excluded males

cat(" ", file=input.file, append=TRUE, sep = "\n")

cat(0, file=input.file, append=TRUE, sep = "\n") #Number of offspring with known excluded maternity

#Offspring ID, number of excluded females, the IDs of excluded females

cat(" ", file=input.file, append=TRUE, sep = "\n")

cat(0, file=input.file, append=TRUE, sep = "\n") #Number of offspring with known excluded paternal sibships

#Offspring ID, number of excluded paternal sibships, the IDs of excluded offspring

cat(" ", file=input.file, append=TRUE, sep = "\n")

cat(0, file=input.file, append=TRUE, sep = "\n") #Number of offspring with known excluded maternal sibships

#Offspring ID, number of excluded maternal sibships, the IDs of excluded offspring

system(paste("Colony2p.exe IFN:", input.file, sep=""))

results<-read.delim(file=paste(filename,".BestCluster", sep=""), header=T, sep="")

male.matrix<-as.data.frame.matrix(table(results$FatherID, results$MotherID))

male.matrix$male_matings<-rowSums(male.matrix>0)

male.matrix<-male.matrix[!male.matrix$male_matings==0,]

mal<-data.frame(table(male.matrix$male_matings))

dimnames(mal)[[2]]<-c("N_matings", "N_indiv")

mal$sex<-c(rep("males", nrow(mal)))

female.matrix<-as.data.frame.matrix(table(results$MotherID, results$FatherID))

female.matrix$female_matings<-rowSums(female.matrix>0)

female.matrix<-female.matrix[!female.matrix$female_matings==0,]

femal<-data.frame(table(female.matrix$female_matings))

dimnames(femal)[[2]]<-c("N_matings", "N_indiv")

femal$sex<-c(rep("females", nrow(femal)))

total.males<-sum(mal$N_indiv)

total.females<-sum(femal$N_indiv)

poly.males<-subset(mal, !mal$N_matings==1)

poly.females<-subset(femal, !femal$N_matings==1)

Mult.mating.males<-sum(poly.males$N_indiv)

Mult.mating.females<-sum(poly.females$N_indiv)

Polygamy.rate.males<-Mult.mating.males/total.males

Polygamy.rate.females<-Mult.mating.females/total.females

mal$N_matings<-as.numeric(mal$N_matings)

mal$N_indiv<-as.numeric(mal$N_indiv)

total.matings.males<-sum(mal$N_matings*mal$N_indiv)

femal$N_matings<-as.numeric(femal$N_matings)

femal$N_indiv<-as.numeric(femal$N_indiv)

total.matings.females<-sum(femal$N_matings*femal$N_indiv)

Average.mating.males<-total.matings.males/total.males

Average.mating.females<-total.matings.females/total.females

Ne<-scan(paste(filename,".Ne",sep=""), what=list(character()))

cat(filename,i,total.males,total.females,Polygamy.rate.males,Polygamy.rate.females,Average.mating.males,Average.mating.females,file=outputfile,

Ne[[1]][18],Ne[[1]][21],Ne[[1]][24],append=TRUE,sep = ",",fill=TRUE)

file.remove(list.files(pattern=filename))

}

}

#------------------------------------------------

# Analyses for subsampling the number of markers

#------------------------------------------------

inputfile<-read.csv(file="inputfile.csv", header=T, sep=";")

outputfile<-"outputfile.txt"

cat("filename","n_loci","totmal","totfem","Polymal","Polyfem","Avgemal","Avgefem","Ne","Ne_min","Ne_max",file=outputfile,append=TRUE,sep = ",",fill=TRUE)

for (i in 1:((ncol(inputfile)-2)/2)){

nloci<-i

for (j in 1:10) { #Set the desired number of replicates for each number of markers

colsample<-sample(seq(from=3, to=ncol(inputfile), by=2), i)

selection<-inputfile[c(1,2, rbind(colsample,colsample+1))]

offspring<-subset(selection, selection$sex_stage=="offspring")

offspring$drop<-rowSums(offspring[,3:ncol(offspring)])

offspring<-subset(offspring, offspring$drop>0)

offspring<-offspring[,1:(ncol(offspring)-1)]

offspring<-offspring[c(-2)]

cmales<-subset(selection, selection$sex_stage=="male")

cmales<-cmales[c(-2)]

cfemales<-subset(selection, selection$sex_stage=="female")

cfemales<-cfemales[c(-2)]

filename<-paste("analysis",i,"loci",j,sep="_")

input.file<-paste(filename,".dat", sep="")

options(width=1000)

cat(filename, file=input.file, append=TRUE, sep = "\n")

cat(filename, file=input.file, append=TRUE, sep = "\n")

cat(length(offspring$ID), file=input.file, append=TRUE, sep = "\n")

cat((ncol(offspring)-1)/2, file=input.file, append=TRUE, sep = "\n")

cat(sample(100000, 1), file=input.file, append=TRUE, sep = "\n")

cat(0, file=input.file, append=TRUE, sep = "\n") #0/1=Not updating/updating allele frequency

cat(2, file=input.file, append=TRUE, sep = "\n") #2/1=Dioecious/Monoecious species

cat(0, file=input.file, append=TRUE, sep = "\n") #0/1=Inbreeding absent/present

cat(0, file=input.file, append=TRUE, sep = "\n") #0/1=Diploid species/HaploDiploid species

cat("0 0", file=input.file, append=TRUE, sep = "\n") #0/1=Polygamy/Monogamy for males & females

cat(0, file=input.file, append=TRUE, sep = "\n") #0/1 = Clone inference = No/Yes

cat(0, file=input.file, append=TRUE, sep = "\n") #0/1=Scale full sibship=No/Yes

cat("1 1 1", file=input.file, append=TRUE, sep = "\n") #0/1/2/3/4=No/Weak/Medium/Strong sibship prior; 4=Optimal sibship prior for Ne

cat(0, file=input.file, append=TRUE, sep = "\n") #0/1=Unknown/Known population allele frequency

cat(1, file=input.file, append=TRUE, sep = "\n") #Number of runs

cat(2, file=input.file, append=TRUE, sep = "\n") #1/2/3/4 = Short/Medium/Long/VeryLong run

cat(0, file=input.file, append=TRUE, sep = "\n") #0/1=Monitor method by Iterate#/Time in second

cat("100000", file=input.file, append=TRUE, sep = "\n") #Monitor interval in Iterate# / in seconds

cat(0, file=input.file, append=TRUE, sep = "\n") #0/1=DOS/Windows version

cat(1, file=input.file, append=TRUE, sep = "\n") #0/1/2=Pair-Likelihood-Score(PLS)/Full-Likelihood(FL)/FL-PLS-combined(FPLS) method

cat(1, file=input.file, append=TRUE, sep = "\n") #0/1/2/3=Low/Medium/High/VeryHigh precision

cat(" ", file=input.file, append=TRUE, sep = "\n")

cat(names(offspring)[seq(from=2, to=ncol(offspring), by=2)], file=input.file, append=TRUE, sep = ",", fill=TRUE)

cat(rep(0,ncol(offspring)/2), file=input.file, append=TRUE, sep = ",", fill=TRUE) #Marker types, 0/1=Codominant/Dominant

cat(rep(0.05,(ncol(offspring)-1)/2), file=input.file, append=TRUE, sep = ",", fill=TRUE) #Allelic dropout rate at each locus

cat(rep(0.05,(ncol(offspring)-1)/2), file=input.file, append=TRUE, sep = ",", fill=TRUE) #Other typing error rate at each locus

cat(" ", file=input.file, append=TRUE, sep = "\n")

for (k in 1:nrow(offspring)){

cat(as.matrix(offspring[k,]), file=input.file, append=TRUE, sep = " ", fill=TRUE)

}

cat(" ", file=input.file, append=TRUE, sep = "\n")

cat("0.66 0.61", file=input.file, append=TRUE, sep = " ", fill=TRUE) #probabilities that the father and mother of an offspring are included in candidates

cat(c(nrow(cmales),nrow(cfemales)), file=input.file, append=TRUE, sep = " ", fill=TRUE)

cat(" ", file=input.file, append=TRUE, sep = "\n")

for (l in 1:nrow(cmales)){

cat(as.matrix(cmales[l,]), file=input.file, append=TRUE, sep = " ", fill=TRUE)

}

cat(" ", file=input.file, append=TRUE, sep = "\n")

for (m in 1:nrow(cfemales)){

cat(as.matrix(cfemales[m,]), file=input.file, append=TRUE, sep = " ", fill=TRUE)

}

cat(" ", file=input.file, append=TRUE, sep = "\n")

cat(0, file=input.file, append=TRUE, sep = "\n") #Number of offspring with known paternity

#IDs of known offspring-father dyad (if any)

cat(" ", file=input.file, append=TRUE, sep = "\n")

cat(5, file=input.file, append=TRUE, sep = "\n") #Number of offspring with known maternity

cat("GSC081 BC09421", file=input.file, append=TRUE, sep = "\n") #IDs of known offspring-mother dyad (if any)

cat("GSC082 BC09421", file=input.file, append=TRUE, sep = "\n") #IDs of known offspring-mother dyad (if any)

cat("GSC083 BC09421", file=input.file, append=TRUE, sep = "\n") #IDs of known offspring-mother dyad (if any)

cat("GSC084 BC09421", file=input.file, append=TRUE, sep = "\n") #IDs of known offspring-mother dyad (if any)

cat("GSC085 BC09421", file=input.file, append=TRUE, sep = "\n") #IDs of known offspring-mother dyad (if any)

cat(" ", file=input.file, append=TRUE, sep = "\n")

cat(0, file=input.file, append=TRUE, sep = "\n") #Number of known paternal sibship

#Size of known paternal sibship, and IDs of offspring in the sibship (if any)

cat(" ", file=input.file, append=TRUE, sep = "\n")

cat(0, file=input.file, append=TRUE, sep = "\n") #Number of known maternal sibship

#Size of known maternal sibship, and IDs of offspring in the sibship (if any)

cat(" ", file=input.file, append=TRUE, sep = "\n")

cat(0, file=input.file, append=TRUE, sep = "\n") #Number of offspring with known excluded paternity

#Offspring ID, number of excluded males, the IDs of excluded males

cat(" ", file=input.file, append=TRUE, sep = "\n")

cat(0, file=input.file, append=TRUE, sep = "\n") #Number of offspring with known excluded maternity

#Offspring ID, number of excluded females, the IDs of excluded females

cat(" ", file=input.file, append=TRUE, sep = "\n")

cat(0, file=input.file, append=TRUE, sep = "\n") #Number of offspring with known excluded paternal sibships

#Offspring ID, number of excluded paternal sibships, the IDs of excluded offspring

cat(" ", file=input.file, append=TRUE, sep = "\n")

cat(0, file=input.file, append=TRUE, sep = "\n") #Number of offspring with known excluded maternal sibships

#Offspring ID, number of excluded maternal sibships, the IDs of excluded offspring

system(paste("Colony2p.exe IFN:", input.file, sep=""))

results<-read.delim(file=paste(filename,".BestCluster", sep=""), header=T, sep="")

male.matrix<-as.data.frame.matrix(table(results$FatherID, results$MotherID))

male.matrix$male_matings<-rowSums(male.matrix>0)

male.matrix<-male.matrix[!male.matrix$male_matings==0,]

mal<-data.frame(table(male.matrix$male_matings))

dimnames(mal)[[2]]<-c("N_matings", "N_indiv")

mal$sex<-c(rep("males", nrow(mal)))

female.matrix<-as.data.frame.matrix(table(results$MotherID, results$FatherID))

female.matrix$female_matings<-rowSums(female.matrix>0)

female.matrix<-female.matrix[!female.matrix$female_matings==0,]

femal<-data.frame(table(female.matrix$female_matings))

dimnames(femal)[[2]]<-c("N_matings", "N_indiv")

femal$sex<-c(rep("females", nrow(femal)))

total.males<-sum(mal$N_indiv)

total.females<-sum(femal$N_indiv)

poly.males<-subset(mal, !mal$N_matings==1)

poly.females<-subset(femal, !femal$N_matings==1)

Mult.mating.males<-sum(poly.males$N_indiv)

Mult.mating.females<-sum(poly.females$N_indiv)

Polygamy.rate.males<-Mult.mating.males/total.males

Polygamy.rate.females<-Mult.mating.females/total.females

mal$N_matings<-as.numeric(mal$N_matings)

mal$N_indiv<-as.numeric(mal$N_indiv)

total.matings.males<-sum(mal$N_matings*mal$N_indiv)

femal$N_matings<-as.numeric(femal$N_matings)

femal$N_indiv<-as.numeric(femal$N_indiv)

total.matings.females<-sum(femal$N_matings*femal$N_indiv)

Average.mating.males<-total.matings.males/total.males

Average.mating.females<-total.matings.females/total.females

Ne<-scan(paste(filename,".Ne",sep=""), what=list(character()))

cat(filename,nloci,total.males,total.females,Polygamy.rate.males,Polygamy.rate.females,Average.mating.males,Average.mating.females,file=outputfile,

Ne[[1]][18],Ne[[1]][21],Ne[[1]][24],append=TRUE,sep = ",",fill=TRUE)

file.remove(list.files(pattern=filename))

}

}

#----------------------------------------------------

# Analyses for subsampling the offspring sample size

#----------------------------------------------------

inputfile<-read.csv(file="inputfile.csv", header=T, sep=";")

outputfile<-"outputfile.txt"

cat("filename","samplesize","totmal","totfem","Polymal","Polyfem","Avgemal","Avgefem","Ne","Ne_min","Ne_max",file=outputfile,append=TRUE,sep = ",",fill=TRUE)

offspring<-subset(inputfile, inputfile$sex_stage=="offspring")

offspring<-offspring[c(-2)]

cmales<-subset(inputfile, inputfile$sex_stage=="male")

cmales<-cmales[c(-2)]

cfemales<-subset(inputfile, inputfile$sex_stage=="female")

cfemales<-cfemales[c(-2)]

for (i in c(10,20,30,40,60)){ #Set the desired sample sizes to explore

samplesize<-i

for (j in 1:10) { #Set the desired number of replicates for each sample size

rowsample<-offspring[sample(1:nrow(offspring), i, replace=FALSE),]

filename<-paste("analysis",i,"offspring",j,sep="_")

input.file<-paste(filename,".dat", sep="")

options(width=1000)

cat(filename, file=input.file, append=TRUE, sep = "\n")

cat(filename, file=input.file, append=TRUE, sep = "\n")

cat(length(rowsample$ID), file=input.file, append=TRUE, sep = "\n")

cat((ncol(rowsample)-1)/2, file=input.file, append=TRUE, sep = "\n")

cat(sample(100000, 1), file=input.file, append=TRUE, sep = "\n")

cat(0, file=input.file, append=TRUE, sep = "\n") #0/1=Not updating/updating allele frequency

cat(2, file=input.file, append=TRUE, sep = "\n") #2/1=Dioecious/Monoecious species

cat(0, file=input.file, append=TRUE, sep = "\n") #0/1=Inbreeding absent/present

cat(0, file=input.file, append=TRUE, sep = "\n") #0/1=Diploid species/HaploDiploid species

cat("0 0", file=input.file, append=TRUE, sep = "\n") #0/1=Polygamy/Monogamy for males & females

cat(0, file=input.file, append=TRUE, sep = "\n") #0/1 = Clone inference = No/Yes

cat(0, file=input.file, append=TRUE, sep = "\n") #0/1=Scale full sibship=No/Yes

cat("1 1 1", file=input.file, append=TRUE, sep = "\n") #0/1/2/3/4=No/Weak/Medium/Strong sibship prior; 4=Optimal sibship prior for Ne

cat(0, file=input.file, append=TRUE, sep = "\n") #0/1=Unknown/Known population allele frequency

cat(1, file=input.file, append=TRUE, sep = "\n") #Number of runs

cat(2, file=input.file, append=TRUE, sep = "\n") #1/2/3/4 = Short/Medium/Long/VeryLong run

cat(0, file=input.file, append=TRUE, sep = "\n") #0/1=Monitor method by Iterate#/Time in second

cat("100000", file=input.file, append=TRUE, sep = "\n") #Monitor interval in Iterate# / in seconds

cat(0, file=input.file, append=TRUE, sep = "\n") #0/1=DOS/Windows version

cat(1, file=input.file, append=TRUE, sep = "\n") #0/1/2=Pair-Likelihood-Score(PLS)/Full-Likelihood(FL)/FL-PLS-combined(FPLS) method

cat(1, file=input.file, append=TRUE, sep = "\n") #0/1/2/3=Low/Medium/High/VeryHigh precision

cat(" ", file=input.file, append=TRUE, sep = "\n")

cat(names(rowsample)[seq(from=2, to=ncol(rowsample), by=2)], file=input.file, append=TRUE, sep = ",", fill=TRUE)

cat(rep(0,ncol(rowsample)/2), file=input.file, append=TRUE, sep = ",", fill=TRUE) #Marker types, 0/1=Codominant/Dominant

cat(rep(0.05,(ncol(rowsample)-1)/2), file=input.file, append=TRUE, sep = ",", fill=TRUE) #Allelic dropout rate at each locus

cat(rep(0.05,(ncol(rowsample)-1)/2), file=input.file, append=TRUE, sep = ",", fill=TRUE) #Other typing error rate at each locus

cat(" ", file=input.file, append=TRUE, sep = "\n")

for (k in 1:nrow(rowsample)){

cat(as.matrix(rowsample[k,]), file=input.file, append=TRUE, sep = " ", fill=TRUE)

}

cat(" ", file=input.file, append=TRUE, sep = "\n")

cat("0.66 0.61", file=input.file, append=TRUE, sep = " ", fill=TRUE) #probabilities that the father and mother of an offspring are included in candidates

cat(c(nrow(cmales),nrow(cfemales)), file=input.file, append=TRUE, sep = " ", fill=TRUE)

cat(" ", file=input.file, append=TRUE, sep = "\n")

for (l in 1:nrow(cmales)){

cat(as.matrix(cmales[l,]), file=input.file, append=TRUE, sep = " ", fill=TRUE)

}

cat(" ", file=input.file, append=TRUE, sep = "\n")

for (m in 1:nrow(cfemales)){

cat(as.matrix(cfemales[m,]), file=input.file, append=TRUE, sep = " ", fill=TRUE)

}

cat(" ", file=input.file, append=TRUE, sep = "\n")

cat(0, file=input.file, append=TRUE, sep = "\n") #Number of offspring with known paternity

#IDs of known offspring-father dyad (if any)

cat(" ", file=input.file, append=TRUE, sep = "\n")

cat(0, file=input.file, append=TRUE, sep = "\n") #Number of offspring with known maternity

#IDs of known offspring-mother dyad (if any)

cat(" ", file=input.file, append=TRUE, sep = "\n")

cat(0, file=input.file, append=TRUE, sep = "\n") #Number of known paternal sibship

#Size of known paternal sibship, and IDs of offspring in the sibship (if any)

cat(" ", file=input.file, append=TRUE, sep = "\n")

cat(0, file=input.file, append=TRUE, sep = "\n") #Number of known maternal sibship

#Size of known maternal sibship, and IDs of offspring in the sibship (if any)

cat(" ", file=input.file, append=TRUE, sep = "\n")

cat(0, file=input.file, append=TRUE, sep = "\n") #Number of offspring with known excluded paternity

#Offspring ID, number of excluded males, the IDs of excluded males

cat(" ", file=input.file, append=TRUE, sep = "\n")

cat(0, file=input.file, append=TRUE, sep = "\n") #Number of offspring with known excluded maternity

#Offspring ID, number of excluded females, the IDs of excluded females

cat(" ", file=input.file, append=TRUE, sep = "\n")

cat(0, file=input.file, append=TRUE, sep = "\n") #Number of offspring with known excluded paternal sibships

#Offspring ID, number of excluded paternal sibships, the IDs of excluded offspring

cat(" ", file=input.file, append=TRUE, sep = "\n")

cat(0, file=input.file, append=TRUE, sep = "\n") #Number of offspring with known excluded maternal sibships

#Offspring ID, number of excluded maternal sibships, the IDs of excluded offspring

system(paste("Colony2p.exe IFN:", input.file, sep=""))

results<-read.delim(file=paste(filename,".BestCluster", sep=""), header=T, sep="")

male.matrix<-as.data.frame.matrix(table(results$FatherID, results$MotherID))

male.matrix$male_matings<-rowSums(male.matrix>0)

male.matrix<-male.matrix[!male.matrix$male_matings==0,]

mal<-data.frame(table(male.matrix$male_matings))

dimnames(mal)[[2]]<-c("N_matings", "N_indiv")

mal$sex<-c(rep("males", nrow(mal)))

female.matrix<-as.data.frame.matrix(table(results$MotherID, results$FatherID))

female.matrix$female_matings<-rowSums(female.matrix>0)

female.matrix<-female.matrix[!female.matrix$female_matings==0,]

femal<-data.frame(table(female.matrix$female_matings))

dimnames(femal)[[2]]<-c("N_matings", "N_indiv")

femal$sex<-c(rep("females", nrow(femal)))

total.males<-sum(mal$N_indiv)

total.females<-sum(femal$N_indiv)

poly.males<-subset(mal, !mal$N_matings==1)

poly.females<-subset(femal, !femal$N_matings==1)

Mult.mating.males<-sum(poly.males$N_indiv)

Mult.mating.females<-sum(poly.females$N_indiv)

Polygamy.rate.males<-Mult.mating.males/total.males

Polygamy.rate.females<-Mult.mating.females/total.females

mal$N_matings<-as.numeric(mal$N_matings)

mal$N_indiv<-as.numeric(mal$N_indiv)

total.matings.males<-sum(mal$N_matings*mal$N_indiv)

femal$N_matings<-as.numeric(femal$N_matings)

femal$N_indiv<-as.numeric(femal$N_indiv)

total.matings.females<-sum(femal$N_matings*femal$N_indiv)

Average.mating.males<-total.matings.males/total.males

Average.mating.females<-total.matings.females/total.females

Ne<-scan(paste(filename,".Ne",sep=""), what=list(character()))

cat(filename,samplesize,total.males,total.females,Polygamy.rate.males,Polygamy.rate.females,Average.mating.males,Average.mating.females,file=outputfile,

Ne[[1]][18],Ne[[1]][21],Ne[[1]][24],append=TRUE,sep = ",",fill=TRUE)

file.remove(list.files(pattern=filename))

}

}

**Appendix S2.** Results of CMR analyses.

**Table S2.1.** Summary table showing the output of the top three ranked models in each species, which cumulated >99% of Corrected Akaike Information Criterion (AICc) weight. Models were named following the parameterization of 1) the temporary emigration/immigration (emi/imm) as dependent (‘Markovian’) or independent (‘Random’) on the last state of the individual, or absent (i.e., fixed to zero, ‘No mov’) and 2) the annual survival rate as sex- (s), time- (t) dependent, or both (s*t), or constant (.). For each model, the table shows the total number of parameters (Num. of params.) of the model, and the estimates (with the 95% CI) of the average probability of survival and temporary emigration/immigration rates of males (*m*) and females (*f*) between consecutive breeding seasons from 2010 to 2015, and the *N*_a_ by sex for each year. Estimates showing unreliably small or large standard errors were considered as non-estimable, and are indicated with a ‘-’. Slight differences between the average of *N*_a_ estimates of the models shown in this table and the estimates shown in Table 1 are caused by the effect of additional models with low AICc weights on model-weighted-average estimates of Table 1. Estimation of all parameters for females of *H. molleri* and *N*_a_ for both sexes of *P. perezi* in 2012 was not attempted due to low recapture rates. For the same reason, * average probabilities of survival of males and females of *P. perezi* between the breeding seasons of 2011-2012 and 2012-2013 could not be distinguished, and so the corresponding survival probabilities for the biannual period 2011-2013 were calculated.

|  |  | ***E. calamita*** | | | ***H. molleri*** | | | ***P. perezi*** | | |
| --- | --- | --- | --- | --- | --- | --- | --- | --- | --- | --- |
|  |  | **1** | **2** | **3** | **1** | **2** | **3** | **1** | **2** | **3** |
| **Model name** |  | Random - S(.) | Random - S(g) | Random - S(t) | No mov - S(.) | No mov - S(t) | Random - S(.) | No mov - S(.) | No mov - S(g) | No mov - S(t) |
| **AICc** |  | -693.83 | -693.29 | -687.79 | -1140.06 | -1133.92 | -1132.82 | 251.75 | 253.78 | 259.61 |
| **AICc Weight** |  | 0.55 | 0.42 | 0.03 | 0.93 | 0.04 | 0.02 | 0.72 | 0.26 | 0.01 |
| **Num. of params.** |  | 75 | 76 | 79 | 25 | 29 | 29 | 50 | 51 | 53 |
| **Average survival 2010-2011** | *m* | 0.61 (0.56-0.66) | 0.6 (0.55-0.66) | 0.66 (0.49-0.8) | 0.15 (0.11-0.22) | 0.17 (0.06-0.4) | 0.16 (0.09-0.27) | 0.31 (0.25-0.38) | 0.28 (0.2-0.38) | 0.36 (0.19-0.56) |
|  | *f* | 0.61 (0.56-0.66) | 0.79 (0.36-0.96) | 0.66 (0.49-0.8) |  |  |  | 0.31 (0.25-0.38) | 0.34 (0.25-0.45) | 0.36 (0.19-0.56) |
| **Average survival 2011-2012** | *m* | 0.61 (0.56-0.66) | 0.6 (0.55-0.66) | 0.52 (0.42-0.63) | 0.15 (0.11-0.22) | 0.04 (0.01-0.21) | 0.16 (0.09-0.27) | 0.31 (0.25-0.38)* | 0.28 (0.2-0.38)*  0.34 (0.25-0.45)* | 0.29 (0.2-0.41)* |
|  | *f* | 0.61 (0.56-0.66) | 0.79 (0.36-0.96) | 0.52 (0.42-0.63) |  |  |  |  |  |  |
| **Average survival 2012-2013** | *m* | 0.61 (0.56-0.66) | 0.6 (0.55-0.66) | 0.7 (0.55-0.82) | 0.15 (0.11-0.22) | 0.24 (0.07-0.59) | 0.16 (0.09-0.27) |  |  |  |
|  | *f* | 0.61 (0.56-0.66) | 0.79 (0.36-0.96) | 0.7 (0.55-0.82) |  |  |  |  |  |  |
| **Average survival 2013-2014** | *m* | 0.61 (0.56-0.66) | 0.6 (0.55-0.66) | 0.6 (0.45-0.73) | 0.15 (0.11-0.22) | 0.21 (0.1-0.39) | 0.16 (0.09-0.27) | 0.31 (0.25-0.38) | 0.28 (0.2-0.38) | 0.34 (0.2-0.52) |
|  | *f* | 0.61 (0.56-0.66) | 0.79 (0.36-0.96) | 0.6 (0.45-0.73) |  |  |  | 0.31 (0.25-0.38) | 0.34 (0.25-0.45) | 0.34 (0.2-0.52) |
| **Average survival 2014-2015** | *m* | 0.61 (0.56-0.66) | 0.6 (0.55-0.66) | 0.69 (0-1) | 0.15 (0.11-0.22) | 0.15 (0.07-0.29) | 0.16 (0.09-0.27) | 0.31 (0.25-0.38) | 0.28 (0.2-0.38) | 0.26 (0.12-0.46) |
|  | *f* | 0.61 (0.56-0.66) | 0.79 (0.36-0.96) | 0.69 (0-1) |  |  |  | 0.31 (0.25-0.38) | 0.34 (0.25-0.45) | 0.26 (0.12-0.46) |
| **Temporary emi/imm 2010-2011** | *m* | 0.14 (0.04-0.36) | 0.13 (0.04-0.36) | 0.16 (0.05-0.43) | 0 (fixed) | 0 (fixed) | - | 0 (fixed) | 0 (fixed) | 0 (fixed) |
|  | *f* | - | - | - |  |  |  | 0 (fixed) | 0 (fixed) | 0 (fixed) |
| **Temporary emi/imm 2011-2012** | *m* | 0.04 (0-1) | 0.03 (0-1) | - | 0 (fixed) | 0 (fixed) | 0.85 (0.22-0.99) | 0 (fixed) | 0 (fixed) | 0 (fixed) |
|  | *f* | 0.81 (0.37-0.97) | 0.85 (0.45-0.97) | 0.78 (0.32-0.96) |  |  |  | 0 (fixed) | 0 (fixed) | 0 (fixed) |
| **Temporary emi/imm 2012-2013** | *m* | 0.09 (0.02-0.27) | 0.08 (0.02-0.27) | 0.1 (0.03-0.3) | 0 (fixed) | 0 (fixed) | 0.08 (0-1) | 0 (fixed) | 0 (fixed) | 0 (fixed) |
|  | *f* | 0.69 (0.35-0.9) | 0.8 (0.46-0.95) | 0.7 (0.36-0.9) |  |  |  | 0 (fixed) | 0 (fixed) | 0 (fixed) |
| **Temporary emi/imm 2013-2014** | *m* | 0.5 (0.38-0.63) | 0.5 (0.37-0.63) | 0.5 (0.35-0.65) | 0 (fixed) | 0 (fixed) | - | 0 (fixed) | 0 (fixed) | 0 (fixed) |
|  | *f* | 0.84 (0.52-0.96) | 0.9 (0.61-0.98) | 0.84 (0.52-0.96) |  |  |  | 0 (fixed) | 0 (fixed) | 0 (fixed) |
| **Temporary emi/imm 2014-2015** | *m* | 0.19 (0.06-0.47) | 0.17 (0.05-0.47) | 0.28 (0-1) | 0 (fixed) | 0 (fixed) | 0.06 (0-1) | 0 (fixed) | 0 (fixed) | 0 (fixed) |
|  | *f* | 0.34 (0.05-0.82) | 0.64 (0.2-0.93) | 0.41 (0-1) |  |  |  | 0 (fixed) | 0 (fixed) | 0 (fixed) |
| **N_a_ 2010** | *m* | 225 (116-539) | 225 (116-539) | 225 (116-539) | 155 (99-293) | 155 (99-293) | 155 (99-293) | 69 (46-132) | 69 (46-132) | 69 (46-132) |
|  | *f* | - | - | - |  |  |  | 68 (36-186) | 68 (36-186) | 68 (36-186) |
| **N_a_ 2011** | *m* | 156 (154-163) | 156 (154-163) | 156 (154-163) | 306 (228-444) | 309 (227-455) | 308 (228-448) | 63 (48-98) | 62 (47-97) | 65 (48-104) |
|  | *f* | 159 (76-440) | 159 (76-440) | 159 (76-440) |  |  |  | 76 (61-106) | 76 (62-107) | 77 (62-108) |
| **N_a_ 2012** | *m* | 128 (84-251) | 128 (84-251) | 120 (93-169) | 134 (42-522) | 53 (20-239) | 39 (17-181) |  |  |  |
|  | *f* | 12 (7-50) | 12 (7-50) | 12 (7-50) |  |  |  |  |  |  |
| **N_a_ 2013** | *m* | 138 (135-146) | 138 (135-146) | 138 (135-146) | 126 (108-158) | 125 (108-157) | 126 (108-158) | 26 (23-36) | 25 (23-36) | 25 (23-36) |
|  | *f* | 43 (34-68) | 43 (34-68) | 43 (34-68) |  |  |  | 27 (23-43) | 28 (23-44) | 27 (23-43) |
| **N_a_ 2014** | *m* | 70 (68-79) | 70 (68-79) | 70 (68-79) | 144 (105-226) | 155 (108-259) | 146 (105-234) | 20 (18-31) | 20 (18-30) | 20 (18-31) |
|  | *f* | 16 (12-47) | 16 (12-47) | 16 (12-47) |  |  |  | 11 (9-24) | 11 (9-25) | 11 (9-26) |
| **N_a_ 2015** | *m* | 162 (160-169) | 162 (160-169) | 162 (160-169) | 49 (39-78) | 49 (39-81) | 49 (39-81) | - | - | - |
|  | *f* | 125 (94-194) | 125 (94-194) | 125 (94-194) |  |  |  | 11 (10-23) | 11 (10-23) | 11 (10-22) |

**Table S2.2.** Standardized log-odds-ratio (LOR) chi square (χ^2^) statistics for ‘transience’ and ‘trap-dependence’ effects and the associated two-sided *p*-values obtained for each sex of each species in each year with a minimum of three (‘transience’) or four (‘trap-dependence’) CMR sessions. Groups for which data were insufficient to perform the tests are indicated with a ‘-’. Significant results are marked in bold.

| **Species** | **Year** | **Sex** | **Test for ‘transience’** | | **Test for ‘trap-dependence’** | |
| --- | --- | --- | --- | --- | --- | --- |
|  |  |  | **χ^2^** | ***p*** | **χ^2^** | ***p*** |
|  |  |  |  |  |  |  |
| ***E. calamita*** | 2010 | males | - | - | - | - |
|  |  | females | - | - | - | - |
|  | 2011 | males | **2.715** | ***0.007*** | -1.184 | *0.237* |
|  |  | females | 0.966 | *0.334* | - | - |
|  | 2012 | males | - | - | - | - |
|  |  | females | - | - | - | - |
|  | 2013 | males | 1.466 | *0.143* | -1.441 | *0.150* |
|  |  | females | 0.416 | *0.677* | 0.000 | *1.000* |
|  | 2014 | males | -0.147 | *0.884* | - | - |
|  |  | females | -0.549 | *0.583* | - | - |
|  | 2015 | males | 0.957 | *0.338* | - | - |
|  |  | females | 0.046 | *0.963* | - | - |
|  |  |  |  |  |  |  |
| ***H. molleri*** | 2010 | males | - | - | - | - |
|  |  | females | - | - | - | - |
|  | 2011 | males | -0.581 | *0.561* | - | - |
|  |  | females | - | - | - | - |
|  | 2012 | males | - | - | - | - |
|  |  | females | - | - | - | - |
|  | 2013 | males | -0.690 | *0.490* | 0.900 | *0.368* |
|  |  | females | - | - | - | - |
|  | 2014 | males | - | - | - | - |
|  |  | females | - | - | - | - |
|  | 2015 | males | - | - | - | - |
|  |  | females | - | - | - | - |
|  |  |  |  |  |  |  |
| ***P. perezi*** | 2010 | males | -0.550 | *0.582* | - | - |
|  |  | females | 0.070 | *0.944* | - | - |
|  | 2011 | males | 0.447 | *0.655* | 0.529 | *0.597* |
|  |  | females | 0.664 | *0.507* | 0.350 | *0.726* |
|  | 2012 | males | - | - | - | - |
|  |  | females | - | - | - | - |
|  | 2013 | males | 1.381 | *0.167* | - | - |
|  |  | females | -0.734 | *0.463* | - | - |
|  | 2014 | males | - | - | - | - |
|  |  | females | - | - | - | - |
|  | 2015 | males | - | - | - | - |
|  |  | females | - | - | - | - |
|  |  |  |  |  |  |  |

**Appendix S3.** Inferred parentages for the tadpole samples (see ID codes in Dryad, doi:10.5061/dryad.2fr3k) of the three species (two different cohorts in the case of *E. calamita*). Inferred parents included in the genotyped candidate parental samples are identified by their ID codes (see data in Dryad, doi:10.5061/dryad.2fr3k). Inferred parents which are not among the genotyped candidate parents are coded with successive numbers (independent among different cohorts) following a * (sires) or a # (dams).

| ***Epidalea calamita* 2013** | | |  | ***Epidalea calamita* 2015** | | |  | ***Hyla molleri*** | | |  | ***Pelophylax perezi*** | | |
| --- | --- | --- | --- | --- | --- | --- | --- | --- | --- | --- | --- | --- | --- | --- |
| **Tadpole** | **Inferred sire** | **Inferred dam** |  | **Tadpole** | **Inferred sire** | **Inferred dam** |  | **Tadpole** | **Inferred sire** | **Inferred dam** |  | **Tadpole** | **Inferred sire** | **Inferred dam** |
|  |  |  |  |  |  |  |  |  |  |  |  |  |  |  |
| GSC049 | *1 | BC09448 |  | GSC492 | BC09723 | BC09715 |  | GSH386 | HY09379 | #1 |  | GS190 | RP09060 | #1 |
| GSC050 | *2 | BC09486 |  | GSC493 | BC09719 | BC09693 |  | GSH387 | *1 | #2 |  | GS191 | *5 | RP09086 |
| GSC051 | BC09328 | BC09467 |  | GSC494 | BC09701 | BC09726 |  | GSH388 | *2 | #3 |  | GS192 | RP09026 | #3 |
| GSC052 | BC09453 | #1 |  | GSC496 | BC09778 | BC09752 |  | GSH389 | *3 | #4 |  | GS193 | *4 | RP09045 |
| GSC053 | *2 | BC09486 |  | GSC498 | BC09778 | BC09728 |  | GSH390 | *4 | #5 |  | GS194 | *14 | RP09037 |
| GSC054 | BC09328 | BC09467 |  | GSC499 | BC09574 | BC09573 |  | GSH391 | *5 | HY09374 |  | GS195 | RP09003 | #13 |
| GSC055 | *2 | BC09486 |  | GSC500 | BC09064 | BC09787 |  | GSH392 | *6 | #6 |  | GS196 | RP09061 | RP09085 |
| GSC056 | BC09328 | BC09467 |  | GSC502 | BC09776 | BC09785 |  | GSH393 | *2 | #1 |  | GS197 | RP09008 | #6 |
| GSC057 | BC09057 | BC09550 |  | GSC504 | BC09488 | BC09779 |  | GSH394 | *1 | #7 |  | GS198 | *1 | RP09030 |
| GSC058 | *2 | BC09486 |  | GSC505 | BC09740 | BC09720 |  | GSH395 | *7 | #8 |  | GS199 | *6 | RP09158 |
| GSC059 | *1 | BC09448 |  | GSC506 | BC09488 | BC09779 |  | GSH396 | *8 | #9 |  | GS200 | RP09060 | #1 |
| GSC060 | *3 | BC09426 |  | GSC508 | BC09705 | BC09579 |  | GSH397 | *9 | HY09412 |  | GS201 | *6 | RP09158 |
| GSC061 | *4 | BC09433 |  | GSC510 | BC09719 | BC09693 |  | GSH398 | *10 | #7 |  | GS202 | *13 | RP09171 |
| GSC062 | BC09057 | BC09550 |  | GSC511 | BC09705 | BC09579 |  | GSH399 | HY09394 | #10 |  | GS203 | *18 | RP09067 |
| GSC063 | BC09141 | BC09429 |  | GSC512 | BC09738 | BC09762 |  | GSH400 | *11 | #11 |  | GS204 | *14 | #2 |
| GSC064 | BC09453 | #1 |  | GSC514 | BC09778 | BC09752 |  | GSH401 | HY09372 | #12 |  | GS205 | *4 | #9 |
| GSC065 | *1 | BC09448 |  | GSC516 | BC09738 | BC09762 |  | GSH402 | *6 | HY09374 |  | GS206 | RP09006 | #2 |
| GSC066 | BC09453 | #1 |  | GSC517 | BC09371 | BC09787 |  | GSH403 | HY09334 | #13 |  | GS207 | *7 | RP09033 |
| GSC067 | BC09141 | BC09429 |  | GSC518 | BC09724 | BC09697 |  | GSH404 | *9 | #9 |  | GS208 | RP09064 | RP09059 |
| GSC068 | BC09057 | BC09550 |  | GSC519 | BC09738 | BC09762 |  | GSH405 | *12 | #14 |  | GS209 | *11 | RP09067 |
| GSC069 | *3 | BC09426 |  | GSC520 | BC09738 | BC09762 |  | GSH406 | HY09399 | #15 |  | GS210 | RP09060 | #1 |
| GSC070 | BC09328 | BC09467 |  | GSC521 | BC09724 | BC09697 |  | GSH407 | HY09334 | #16 |  | GS211 | RP09005 | RP09066 |
| GSC071 | *4 | BC09433 |  | GSC522 | BC09179 | BC09430 |  | GSH408 | *13 | #17 |  | GS212 | *2 | #2 |
| GSC072 | BC09141 | BC09429 |  | GSC523 | BC09179 | BC09430 |  | GSH409 | *11 | #14 |  | GS213 | *8 | RP09067 |
| GSC073 | BC09328 | BC09467 |  | GSC524 | BC09744 | BC09755 |  | GSH410 | *14 | #11 |  | GS214 | RP09026 | #3 |
| GSC074 | *2 | BC09486 |  | GSC526 | BC09744 | BC09755 |  | GSH411 | *15 | #18 |  | GS215 | RP09026 | #3 |
| GSC075 | *4 | BC09433 |  | GSC528 | BC09405 | BC09790 |  | GSH412 | *16 | #19 |  | GS216 | *8 | #6 |
| GSC076 | *2 | BC09486 |  | GSC529 | BC09405 | BC09790 |  | GSH413 | HY09404 | #20 |  | GS217 | *10 | #10 |
| GSC077 | BC09453 | #1 |  | GSC530 | BC09179 | BC09430 |  | GSH414 | HY09404 | #21 |  | GS218 | RP09024 | RP09053 |
| GSC078 | *3 | BC09426 |  | GSC532 | BC09405 | BC09790 |  | GSH415 | *17 | #22 |  | GS219 | *15 | RP09050 |
| GSC079 | *1 | BC09448 |  | GSC534 | BC09731 | BC09733 |  | GSH416 | *18 | #11 |  | GS220 | RP09006 | #2 |
| GSC080 | *1 | BC09448 |  | GSC535 | BC09179 | BC09430 |  | GSH417 | HY09377 | #23 |  | GS221 | RP09026 | RP09040 |
| GSC081 | *5 | BC09421 |  | GSC536 | BC09405 | BC09790 |  | GSH418 | HY09379 | #1 |  | GS222 | *1 | #4 |
| GSC082 | *5 | BC09421 |  | GSC537 | BC09405 | BC09790 |  | GSH419 | *19 | #5 |  | GS223 | *6 | RP09158 |
| GSC083 | *5 | BC09421 |  | GSC538 | BC09744 | BC09755 |  | GSH420 | *20 | #24 |  | GS224 | *10 | #3 |
| GSC084 | *5 | BC09421 |  | GSC539 | BC09179 | BC09430 |  | GSH421 | *21 | #25 |  | GS225 | RP09012 | RP09152 |
| GSC085 | *5 | BC09421 |  | GSC540 | BC09744 | BC09755 |  | GSH422 | *10 | HY09418 |  | GS226 | *15 | #12 |
| GSC086 | *6 | #2 |  | GSC541 | BC09744 | BC09755 |  | GSH423 | *19 | #8 |  | GS227 | RP09064 | RP09059 |
| GSC087 | *7 | #3 |  | GSC542 | BC09105 | #1 |  | GSH424 | *16 | #4 |  | GS228 | RP09015 | RP09040 |
| GSC088 | *6 | #2 |  | GSC544 | *1 | BC09604 |  | GSH425 | *22 | #26 |  | GS229 | RP09061 | RP09085 |
| GSC089 | BC09110 | BC09451 |  | GSC546 | *1 | BC09604 |  | GSH426 | *23 | #27 |  | GS230 | *2 | #2 |
| GSC090 | BC09110 | BC09451 |  | GSC547 | BC09694 | BC09708 |  | GSH427 | HY09406 | #28 |  | GS231 | *8 | RP09063 |
| GSC091 | *6 | #2 |  | GSC548 | BC09694 | BC09708 |  | GSH428 | *24 | #1 |  | GS232 | RP09061 | RP09051 |
| GSC092 | *5 | BC09575 |  | GSC550 | BC09694 | BC09708 |  | GSH429 | *20 | #1 |  | GS233 | *2 | #2 |
| GSC093 | BC09443 | BC09575 |  | GSC552 | *1 | BC09604 |  | GSH430 | *14 | #10 |  | GS234 | RP09060 | #1 |
| GSC094 | *6 | #2 |  | GSC553 | BC09694 | BC09708 |  | GSH431 | HY09371 | #29 |  | GS235 | *17 | RP09030 |
| GSC095 | BC09110 | BC09451 |  | GSC554 | *1 | BC09604 |  | GSH432 | HY09367 | #30 |  | GS236 | *12 | RP09016 |
| GSC096 | BC09110 | BC09451 |  | GSC556 | BC09694 | BC09708 |  | GSH433 | HY09393 | #22 |  | GS237 | *19 | RP09042 |
| GSC097 | BC09110 | BC09451 |  | GSC557 | BC09694 | BC09708 |  | GSH434 | *20 | #11 |  | GS238 | RP09060 | #1 |
| GSC098 | BC09334 | #4 |  | GSC558 | *1 | BC09604 |  | GSH435 | HY09377 | #31 |  | GS239 | *9 | RP09085 |
| GSC099 | BC09317 | BC09591 |  | GSC559 | BC09153 | BC09573 |  | GSH436 | *25 | #4 |  | GS240 | *11 | RP09171 |
| GSC100 | BC09317 | BC09591 |  | GSC560 | *1 | BC09604 |  | GSH437 | *26 | #4 |  | GS241 | *9 | RP09085 |
| GSC101 | *7 | #1 |  | GSC561 | *1 | BC09604 |  | GSH438 | *27 | #32 |  | GS242 | *2 | #2 |
| GSC102 | BC09334 | #4 |  | GSC562 | BC09594 | BC09571 |  | GSH439 | *11 | #19 |  | GS243 | RP09060 | #1 |
| GSC103 | BC09036 | BC09593 |  | GSC564 | BC09594 | BC09571 |  | GSH440 | *11 | #11 |  | GS244 | *13 | #6 |
| GSC104 | BC09414 | BC09476 |  | GSC565 | BC09776 | #2 |  | GSH441 | *27 | #29 |  | GS245 | RP09061 | RP09085 |
| GSC105 | BC09440 | #5 |  | GSC566 | *2 | #3 |  | GSH442 | HY09371 | #33 |  | GS246 | *3 | #5 |
| GSC106 | BC09334 | #4 |  | GSC568 | BC09233 | BC09426 |  | GSH443 | *28 | HY09414 |  | GS247 | RP09006 | #2 |
| GSC107 | BC09334 | #4 |  | GSC570 | BC09589 | #4 |  | GSH444 | HY09292 | HY09405 |  | GS248 | RP09060 | #1 |
| GSC108 | BC09057 | #6 |  | GSC572 | BC09551 | #5 |  | GSH445 | HY09372 | #17 |  | GS249 | *12 | #11 |
| GSC109 | BC09097 | BC09311 |  | GSC574 | BC09233 | BC09426 |  | GSH446 | HY09215 | #34 |  | GS250 | RP09012 | RP09152 |
| GSC110 | BC09440 | #5 |  | GSC575 | *3 | BC09426 |  | GSH447 | *19 | #28 |  | GS252 | RP09006 | #14 |
| GSC111 | BC09440 | #5 |  | GSC576 | BC09771 | BC09755 |  | GSH448 | HY09396 | #22 |  | GS253 | *11 | RP09067 |
| GSC112 | BC09036 | BC09593 |  | GSC578 | BC09105 | #1 |  | GSH449 | HY09338 | #9 |  | GS254 | RP09008 | #6 |
| GSC113 | BC09097 | BC09311 |  | GSC580 | BC09163 | BC09585 |  | GSH450 | HY09372 | #35 |  | GS255 | RP09055 | #8 |
| GSC114 | BC09424 | BC09398 |  | GSC582 | BC09163 | BC09585 |  | GSH451 | HY09399 | #7 |  | GS256 | RP09058 | #9 |
| GSC115 | BC09036 | BC09708 |  | GSC584 | BC09163 | BC09585 |  | GSH452 | HY09399 | #36 |  | GS257 | *13 | RP09057 |
| GSC116 | *7 | #7 |  | GSC585 | BC09163 | BC09585 |  | GSH453 | HY09394 | #37 |  | GS258 | *16 | RP09171 |
| GSC117 | BC09424 | BC09398 |  | GSC586 | BC09163 | BC09585 |  | GSH454 | HY09400 | #38 |  | GS259 | *15 | RP09036 |
| GSC118 | BC09334 | #4 |  | GSC587 | *1 | #6 |  | GSH455 | HY09379 | #35 |  | GS260 | RP09064 | RP09032 |
| GSC119 | BC09462 | #3 |  | GSC588 | *4 | #5 |  | GSH456 | *24 | #39 |  | GS261 | RP09006 | #14 |
| GSC121 | *8 | BC09575 |  | GSC590 | BC09269 | #7 |  | GSH457 | *29 | #40 |  | GS262 | *4 | #7 |
| GSC122 | BC09424 | BC09398 |  | GSC591 | *4 | #5 |  | GSH458 | *8 | #39 |  | GS263 | RP09061 | RP09051 |
| GSC123 | BC09317 | BC09591 |  |  |  |  |  | GSH459 | *16 | #39 |  | GS264 | RP09058 | #9 |
| GSC124 | BC09036 | BC09593 |  |  |  |  |  | GSH460 | HY09377 | #31 |  | GS265 | RP09049 | RP09180 |
| GSC125 | BC09330 | BC09575 |  |  |  |  |  | GSH461 | *1 | #37 |  | GS266 | *17 | #8 |
| GSC126 | BC09414 | BC09476 |  |  |  |  |  | GSH462 | HY09406 | HY09414 |  | GS267 | RP09131 | RP09066 |
|  |  |  |  |  |  |  |  | GSH463 | *30 | #8 |  | GS268 | RP09013 | RP09057 |
|  |  |  |  |  |  |  |  | GSH464 | *17 | #41 |  | GS269 | *13 | RP09007 |
|  |  |  |  |  |  |  |  | GSH465 | *11 | #38 |  | GS270 | RP09012 | RP09152 |
|  |  |  |  |  |  |  |  | GSH466 | *20 | #24 |  | GS271 | *3 | #5 |
|  |  |  |  |  |  |  |  | GSH467 | HY09337 | HY09412 |  | GS272 | RP09039 | RP09063 |
|  |  |  |  |  |  |  |  | GSH468 | *26 | #42 |  | GS273 | *3 | RP09007 |
|  |  |  |  |  |  |  |  | GSH469 | *21 | #5 |  | GS274 | *7 | RP09042 |
|  |  |  |  |  |  |  |  | GSH470 | HY09400 | #2 |  | GS275 | *8 | RP09067 |
|  |  |  |  |  |  |  |  | GSH471 | *1 | #43 |  | GS276 | RP09012 | RP09152 |
|  |  |  |  |  |  |  |  | GSH472 | *6 | #6 |  | GS277 | *20 | RP09007 |
|  |  |  |  |  |  |  |  | GSH473 | *8 | #6 |  | GS278 | *1 | RP09051 |
|  |  |  |  |  |  |  |  | GSH474 | *31 | #3 |  | GS280 | *3 | #5 |
|  |  |  |  |  |  |  |  | GSH475 | *32 | #44 |  | GS281 | RP09006 | #2 |
|  |  |  |  |  |  |  |  | GSH476 | *33 | #5 |  | GS282 | *10 | #3 |
|  |  |  |  |  |  |  |  | GSH477 | *34 | #35 |  | GS283 | *11 | RP09067 |
|  |  |  |  |  |  |  |  | GSH478 | HY09394 | #45 |  | GS284 | RP09061 | RP09051 |
|  |  |  |  |  |  |  |  | GSH479 | HY09330 | #46 |  | GS285 | *18 | RP09063 |
|  |  |  |  |  |  |  |  | GSH480 | *26 | #19 |  |  |  |  |
|  |  |  |  |  |  |  |  | GSH481 | *6 | #27 |  |  |  |  |
